# Optimization of wavy trapezoidal porous cavity containing mixture hybrid nanofluid (water/ethylene glycol Go–Al_2_O_3_) by response surface method

**DOI:** 10.1038/s41598-023-28916-2

**Published:** 2023-01-30

**Authors:** Navid Alipour, Bahram Jafari, Kh. Hosseinzadeh

**Affiliations:** 1 Faculty of Engineering Modern Technologies, Amol University of Special Modern Technologies (AUSMT), Amol, Iran; 2 Department of Mechanical Engineering, Babol Noshirvani University of Technology, Babol, Iran

**Keywords:** Engineering, Mechanical engineering

## Abstract

Increasing thermal performance and preventing heat loss are very important in energy conversion systems, especially for new and complex products that exacerbate this need. Therefore, to solve this challenge, a trapezoidal cavity with a wavy top wall containing water/ethylene glycol GO–Al_2_O_3_ nanofluid is simulated using Galerkin finite element method. The effects of physical parameters affecting thermal performance and fluid flow, including porosity (ℇ), thermal radiation (Rd), magnetic field angle (α), Rayleigh number (Ra) and Hartmann number (Ha), are investigated in the determined ratios. The results of applied boundary conditions showed that the optimal values for Ra, Ha, ℇ, Rd and α are 1214.46, 2.86, 0.63, 0.24 and 59.35, respectively. Considering that changes in radiation have little effect on streamlines and isothermal lines. Optimization by RSM and Taguchi integration resulted in optimal Nu detection. It provided a correlation for the average Nu based on the investigated determinants due to the conflicting influence of the study factors, which finally calculated the highest average Nusselt number of 3.07. Therefore, the ideal design, which is the primary goal of this research, increases the thermal performance.

## Introduction

Analysis of natural convection and fluid flow in cavities with different shapes is one of the most important and widely used topics in engineering and industry. A review of studies in this field shows that the shape of different configurations such as triangular, square, trapezoidal cavities etc., affects heat transfer, fluid flow and energy loss. For example, rounded cavities are used to cooling circuit boards, heat exchangers and solar air heaters. To reduce the amount of lost energy inside a trapezoidal chamber filled with water nanofluid, combined water and convection flow, heat transfer, as well as the magnetic field, were studied by Mondal and Mahapatra^1^. Moreover, they concluded that a rectangular enclosure with small dimensions and a low magnetic field effectively decrease total entropy. Yusuf and Osman^2^ carefully investigated the natural convection in a square cavity in a two-dimensional space with roughness on the vertical wall. The sinusoidal roughness significantly affects the thermal behavior and hydrodynamics of the fluid in question. The biggest reduction in heat transfer was calculated to be 28%. Barnoon et al.^3^ examined the natural convection in a cavity with a non-Newtonian nanofluid including two cylinders inside it under thermal radiation and without it. According to the simulations performed in different conditions, changing the cavity's angle can be effective in heat transfer; also, increasing the cylinders' size according to the cavity's angle can decrease or increase the heat transfer. Ghalambaz et al.^4^ evaluated the ferro-hydrodynamic and magneto-hydrodynamic effects inside a hexagonal chamber using different nanofluids in a non-uniform magnetic field. According to the results, with an increase in the magnetic number, the heat, and mass transfer rate increase, while the increase of Lorentz force decreases the heat transfer rate. Convection flow on the grooves of an open ring inside a cavity containing nanofluid was studied by Aly^5^. According to the results obtained from the concentrated ISPH method, the flow intensity and fluid concentration distribution decrease with a decrease in the Soret number and an increase in the Dufour number. Zhang et al.^6^ analyzed MHD natural convection in two-dimensional and three-dimensional models with thermal radiation impacts. They found that in the three-dimensional case, thermal radiation enhances heat transfer with fluid flow, while the magnetic field has the opposite effect. Sobhani et al.^7^ used the Taguchi test design method to calculate the optimal natural convection values with combined volume radiation on a square cavity with horizontal fins. They utilized the LBM method to solve the governing equations and concluded that the most imperative parameter of the total Nusselt is the Rayleigh number. At the same time, the fin length and height do not greatly affect the total Nusselt number. Natural cause along with surface radiation heat transfer using finite volume method on a square cavity with vertical fins by Moutaouakil et al.^8^ was reviewed. The obtained results show that the air temperature increases with the increase of Rayleigh number, and surface radiation greatly affects the flow in the cavity. Amer Qureshi et al.^9^ simulated heat transfer and fluid flow characteristics in a perforated horizontal channel containing a barrier containing hybrid nanofluid. The results showed that increasing the value of the obstacle radius increases the amount of heat transfer within the channel. Also, the horizontal position of the cylinder affects the heat transfer efficiency. Shekarmaz et al.^10^ modeled the two-phase nanofluid in natural convection for a triangular cavity with a heated corrugated wall. After examining the values of various parameters affecting heat transfer, it was found that the Nusselt number has a direct relationship with the Rayliegh number. In contrast, it has an inverse relationship with the wave number, and the amount of heat transfer has an inverse relationship with the total entropy production. Luo et al.^11^ analyzed the effects of thermal radiation on MHD flow in a cubic cavity with an external magnetic field with different amounts of applied heat. The streamlines and isotherms were analyzed to investigate the heat transfer characteristics. Boukendil et al.^12^ studied natural convection and surface radiation for an asymmetric square cavity containing a heated circular cylinder using the finite volume method. Based on the results the surface radiation applied to the cavity greatly affects the flow structure and heat transfer. The speed of heat transfer, displacement, and entropy generation due to the application of magnetic field and radiation inside a diagonal square cavity containing nanofluid have been investigated by Alnaqi et al.^13^. They presented that higher Nusselt numbers are obtained at high Rayliegh numbers, which means more heat transfer. Moreover, high heat transfer rate values can be achieved by adding the radiation parameter. Simulations for detailed investigation of streamlines and isotherms using dimensionless parameters for a top-walled square cavity heated by magneto-hydrodynamics were performed by Usman et al.^14^. The results indicated that increasing the value of the radiation parameter leads to an increase in Nusselt numbers. Finally, a suggested method for comparing the results is provided. For the first time, Mohammadi and Ganj Alikhan^15^ studied a complex cavity containing a nanofluid that is an electrical conductor. They investigated the effect of magnetic field gradient and optical thickness in this cavity. After applying effective physical parameters, fluid flow and heat transfer characteristics were described. To investigate thermal convection, Abdur Rahim and Al-Sapa^16^ chose a square cavity consisting of an outer rotating cylinder and an inner cross shape. Under the influence of magnetic hydrodynamics, simulations were performed for different parameters. They showed that the Rayleigh number is one of the influential factors in fluid movement and heat transfer, which improves thermal performance in a cavity. Khalil et al.^17^ studied a trapezoidal cavity with a wavy surface in magneto-hydrodynamic conditions and without it to achieve an ideal design with high thermal performance. After applying different conditions and optimizations in terms of average Nusselt number and heat transfer rate, the highest increase in heat transfer and energy increase was obtained in the appropriate geometry under the influence of effective parameters. Kumar Yadav et al.^18^ studied the optimal heat transfer performance for complex geometry in the form of a slotted channel containing nanofluid under magneto-hydrodynamics. The fluid velocity inside the channel is low, and several consequences are applied. The optimal values for the best thermal performance were obtained according to the specified conditions. Using the Computational Fluid Dynamics (CFD) technique, Zakaria Cory et al.^19^ studied a cavity with two round ears containing hybrid nanofluid, and its bottom wall is heated. They found that applying a vertical magnetic field improves the heat transfer and that the smaller radius of the cavity corners can positively affect the heat transfer and irreversibility. investigation of thermal convection coupled with surface radiation for a rotating cavity was performed by Mikhailenko et al.^20^. The chamber in question contains a volumetric heating element. It was found that the total energy transfer increases with surface radiation, and the average temperature of the heater decreases. Also, the amount of heat transfer in the cavity increases with applying the fluctuation frequency of volumetric heat generation. Barnoon et al.^21^ chose a square cavity with inner cylinders to investigate mixed convection and entropy generation. According to the results the reduction of Hartmann's number and Richardson's number increases the amount of heat transfer. In addition, cylinders with angular velocity are also effective in increasing heat transfer. Numerical analysis for a square cavity filled with nanofluid to investigate natural convection was performed by Sreedevi and Reddy^22^ by applying a magnetic field and thermal radiation. The cavity is placed inside two isothermal and adiabatic walls. The results show that the increase in thermal radiation leads to more heat transfer from the hot wall to the cold wall. Sivasankaran et al.^23^ calculated the change of heat transfer rate inside a closed cavity subjected to buoyancy heating and cooling by the finite volume method. With the increase of thermal radiation, the rate of heat change also changes to a non-linear mode. Khan et al.^24^ performed a computational analysis to reduce entropy generation on three porous cavities with different triangular, square, and trapezoidal geometries due to kinetic friction, energy, mass transfer, and porous materials. The results show that the square hole has the lowest total entropy production. Also, the kinetic friction does not greatly affect the total entropy. In order to control the thermal performance inside a wavy porous cavity, a numerical study using partial and semi-active magnetic fields along with hybrid nanofluid flow was carried out by Biswas et al.^25^. Their results showed that using a partial magnetic field to control field variables is more beneficial than the entire magnetic field and has a significant effect on the thermal performance of the entire cavity. Mandal et al.^26^ investigated the bioconvection of a magnetic hydrodynamic mixture, including nanofluid, in a cavity with a wavy wall. The thermal behaviors were evaluated by changing the physical parameters affecting the flow. They concluded that the wavy curved surface increases the heat transfer rate, and bio convection positively affects this increase. Convection flow control and thermal performance were performed using numerical simulation by Biswas et al.^27^. They applied a partial magnetic field to the cavity containing the nanofluid. Their results showed that the convective process could be effectively adjusted by changing and adjusting the partial magnetic field application position, including its width, direction, and intensity. Also, the partial magnetic field reduces heat transfer less than the total magnetic field. Numerical simulation was carried out to investigate the free convection of magnetic hydrodynamics in a wavy inclined cavity containing a hybrid nanofluid by biswas et al.^28^. The results showed that increasing the amplitude of the cavity wave increases the heat transfer. Also, increasing the thermal surface only sometimes positively affects heat transfer. The effect of thermal non-equilibria in a cavity containing nanofluid on the amount of natural convection and entropy production has been done by Tayebi^29^. To compare the thermal irreversibility, introduced a new parameter to evaluate the ratio of entropy production in the fluid to the solid phase. In order to analyze the natural convection flow in an oval cavity with an inner cylinder saturated with nanofluid, thermal non-equilibrium effects have been studied by Tayebi and Chamkha^30^. The obtained results were analyzed based on the conditions and characteristics of porous materials. Numerical simulation to investigate the free convection in a cavity with wavy walls consisting of a solid part, a porous medium part and a nanofluid filled medium part was done by Alsabery et al.^31^. After examining the effective physical parameters, it was found that the high Darcy number plays an important role in thermal imbalance effects. Alsabery et al.^32^ investigated the natural convection in a cavity with wavy nanofluid walls under thermal non-equilibrium conditions. Their results showed that the length of the hot wall, its position, the number of waves on the walls, and the concentration of nanoparticles are among the most critical parameters to control the convection flow and heat transfer. In order to predict the natural heat transfer inside a porous trapezoidal cavity with baffles on its side walls that are in adiabatic conditions, a modeling approach is presented by Zidan et al.^33^. It also evaluates the use of nanomaterials to improve natural convection through economic feasibility. Tayebi et al.^34^ studied natural convection to investigate the effects of thermal non-equilibrium on a porous elliptical cavity containing nanofluid using the finite element method. Their results show, The smaller porous medium and improved thermal conductivity ratio increase the local thermal non-equilibrium (LTNE) effects in the cavity. Biswas et al.^35^ numerically analyzed the fluid containing copper nanoparticles and oxytactic bacteria to investigate the physics of triple convection flow in a new W-shaped porous cavity. Closer shapes, such as square and trapezoidal enclosures, have been used to compare flow physics, heat transfer and mass transfer phenomena. The results showed that the W-shaped cavity has a high heat (Nu) and mass (Sh) transfer rate compared to the traditional square and trapezoidal cavities. Mandal et al.^36^ studied the hydrothermal properties in a non-Darcy porous complex wave chamber saturated with a hybrid nanofluid in the presence of a uniform magnetic field. According to the study's findings, it is clear that the curved wall increases the effective heating surface area, which in turn promotes an increase in thermal energy and, consequently, the size of the average Nu. Mandal et al.^37^ investigated a new M-shaped chamber filled with permeable materials and hybrid nanoparticles suspended in water under a horizontal magnetic field to investigate the heat transfer process in the fluid numerically. By changing the sidewall slope, the number and height of the upper inverted triangular waves under comparable boundary conditions, the effect of the geometrical parameters on the thermal performance have been carefully investigated. Mandal et al.^38^ investigate the mixed magneto-thermal convection in a W-shaped chamber cooled by a horizontal top wall and filled with a hybrid nanofluid saturated in a porous structure. The findings demonstrated that the length of the heating and cooling surface, the volume of fluid within the cavity, and the size of the lower wave height within the W-shaped cavity significantly influence thermal energy transfer. According to the literature review, the analysis of natural heat transfer and fluid flow in the presence of a magnetic field in cavities with different geometries has been investigated in several articles. Considering the various applications of natural heat transfer in fluid-containing cavities in engineering and industry, especially solar collectors, a study was conducted with a new approach in this field. The main goal of this study is to optimize the wavy trapezoidal porous cavity containing the hybrid nanofluid mixture and obtain the optimal values for the mentioned effective physical parameters with and without applying a magnetic field, which leads to an increase in the natural heat transfer performance inside the cavity. In addition, applying the magnetic field with different angles, one of the characteristics of the novelty of the present work, is investigated. The innovation of this study is the design of a unique geometry that has yet to be studied so far and is comparable to the practical design of solar collectors according to the boundary conditions selected in the geometry. Many engineering and industrial systems and devices, including solar collectors, solar dryers, thermal energy storage systems, heat exchangers, etc., can benefit from understanding the design and performance of the current study. The parameters of a suitable and optimal design for a better understanding of the issue, the effect of different values of Rayleigh number, porosity, Hartmann number, and radiation coefficient on the isothermal lines, and streamlines have been investigated. This cavity is porous and contains water-based ethylene glycol hybrid nanofluid GO_Al_2_O_3_, which positively affects the rate of natural convection. GFEM method is used to solve the governing equations. The RSM method is used to optimize and obtain optimal points for the studied parameters along with the Taguchi test design, which can provide reliable results.

## Problem description and governing equations

**Figure 1:**
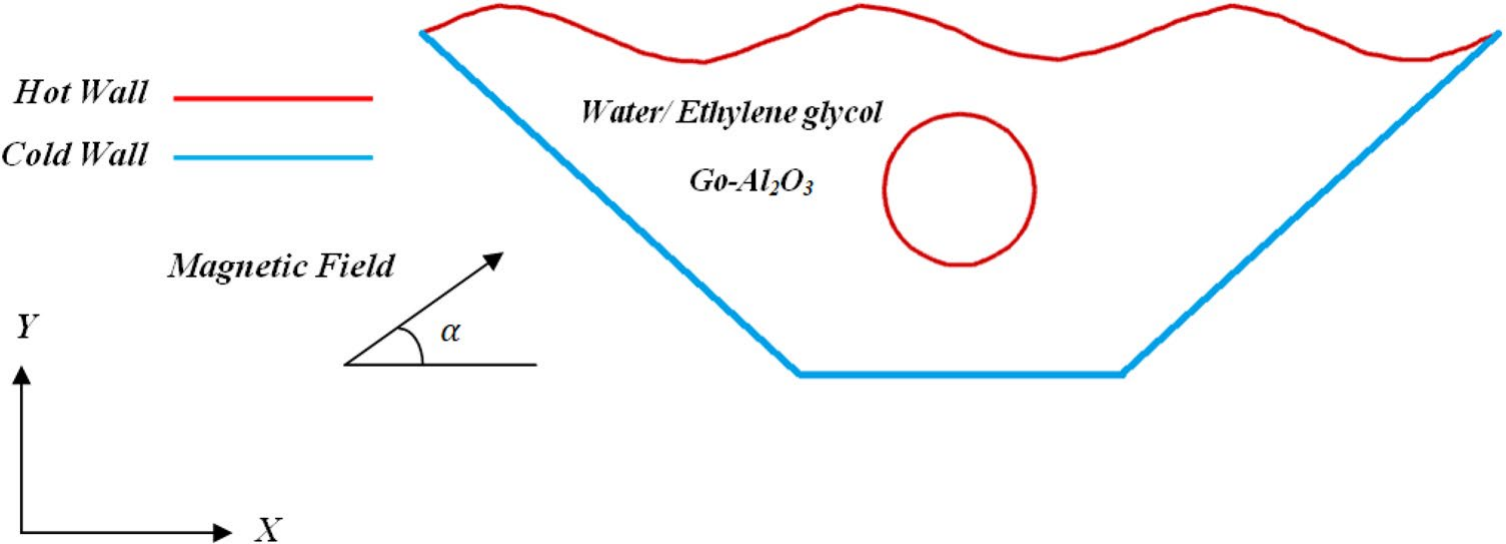
Geometry and boundary conditions.

The schematic of the geometry of the problem under consideration with respect to the boundary conditions is shown in Fig. 1. The selected geometry is a trapezoidal cavity with a wavy top wall and an inner cylindrical heated boundary that has specific dimensions. The cavity under study contains water-based ethylene glycol Go_Al_2_O_3_ hybrid nanofluid. In this study, the hybrid nanofluid is assumed incompressible and newtonian. The flow inside the two-dimensional cavity becomes laminar. Nanoparticles and base fluid are considered in thermal equilibrium, And the volume concentration for nanoparticles is 0.05 or 5%. Darcy's law has been used to model the porous medium^39^. This research has proposed a new solution for numerical simulation of hybrid nanofluid convection heat transfer inside a wavy trapezoidal cavity with the effect of the magnetic field. The Navier–Stokes equations and the conservation of mass equation solve problems related to fluids. It means that the number of unknowns equals the number of equations, and solving the problem theoretically is possible. The continuity and momentum equations (Eqs. 1–3) are shown below to control the fluid flow containing a hybrid nanofluid in the defined boundary domain and to investigate its behavior in two-dimensional mode.

1 $$ \frac{\partial u}{{\partial x}} + \frac{\partial v}{{\partial y}} = 0 $$

2 $$ \frac{{\mu_{nf} }}{K}u - \sigma_{hnf} B_{0}^{2} \left[ {v\left( {\sin \gamma } \right)\left( {\cos \gamma } \right) - u\left( {\sin \gamma } \right)^{2} } \right] = - \frac{\partial p}{{\partial x}} $$

3 $$ \frac{{\mu_{nf} }}{K}v - \sigma_{hnf} B_{0}^{2} \left[ {u\left( {\sin \gamma } \right)\left( {\cos \gamma } \right) - v\left( {\cos \gamma } \right)^{2} } \right] = - \frac{\partial p}{{\partial y}} + g\left( {\rho \beta } \right)_{hnf} \left( {T - T_{c} } \right) $$

The energy equation by performing discretization for thermal analysis is shown below:

4 $$ \begin{aligned} & \frac{1}{\varepsilon }\left( {\left( {\rho c_{p} } \right)_{hnf}^{ - 1} \frac{{\partial q_{r} }}{\partial y} + \left( {u\frac{\partial T}{{\partial x}} + v\frac{\partial T}{{\partial y}}} \right)} \right) = k_{hnf} \left( {\frac{{\partial^{2} T}}{{\partial x^{2} }} + \frac{{\partial^{2} T}}{{\partial y^{2} }}} \right)\left( {\rho c_{p} } \right)_{hnf}^{ - 1} , \\ & q_{r} = \frac{{ - 4\sigma_{e} }}{{3\beta_{R} }}\frac{{\partial T^{4} }}{\partial y},\quad T^{4} \cong 4T_{c}^{3} T - 3T_{c}^{4} \\ \end{aligned} $$

In this study, the thermophysical properties of the hybrid nanofluid were calculated using associated equations^40^. The effective density according to the hybrid nanofluid mixture dispersed in the base fluid is shown in Eq. (5):

5 $$ \rho_{{{\text{hnf}}}} { = }\rho_{{\text{f}}} {(1} - \phi_{2} )\left( {(1 - \phi_{1} ) + \phi_{1} {(}\frac{{\rho_{{{\text{s1}}}} }}{{\rho_{{\text{f}}} }}{)}} \right) + \phi_{2} \rho_{{{\text{s2}}}} $$

The effective volume coefficient of thermal expansion is written as follows^41^:

6 $$ {(}\rho \beta {)}_{{{\text{hnf}}}} { = (}\rho \beta {)}_{{\text{f}}} {(1} - \phi_{2} )((1 - \phi_{1} ) + \phi_{1} \frac{{{(}\rho \beta {)}_{{{\text{s1}}}} }}{{{(}\rho \beta {)}_{{\text{f}}} }}{)} + \phi_{2} {(}\rho \beta {)}_{{{\text{s2}}}} $$

Another parameter that is calculated for the heat evaluation of nanofluid is the effective heat capacity in conditions where there is no pressure gradient, so the heat capacity of nanofluid is calculated from the following equation^42^:

7 $$ {(}\rho {\text{c}}_{{\text{p}}} {)}_{{{\text{hnf}}}} { = (}\rho {\text{c}}_{{\text{p}}} {)}_{{\text{f}}} (1 - \phi_{2} )\left( {(1 - \phi_{1} ) + \phi_{1} \frac{{{(}\rho {\text{c}}_{{\text{p}}} {)}_{{{\text{s1}}}} }}{{{(}\rho {\text{c}}_{{\text{p}}} {)}_{{\text{f}}} }}} \right) + \phi_{2} {(}\rho {\text{c}}_{{\text{p}}} {)}_{{{\text{s2}}}} $$

In addition, the electrical conductivity is calculated by Eq. (8) as^42^:

8 $$ \frac{{\sigma_{{_{{{\text{hnf}}}} }} }}{{\sigma_{{_{{{\text{bf}}}} }} }}{ = 1 + }\frac{{3\phi \left( {\sigma_{1} \phi_{1} + \sigma_{2} \phi_{2} - \sigma_{bf} \left( {\phi_{1} + \phi_{2} } \right)} \right)}}{{\left( {(\sigma_{1} \phi_{1} + \sigma_{2} \phi_{2} + 2\phi \sigma_{bf} ) - \phi \sigma_{bf} ((\sigma_{1} \phi_{1} + \sigma_{2} \phi_{2} ) - \sigma_{bf} (\phi_{1} + \phi_{2} ))} \right)}} $$

The following relationship represents the effective thermal conductivity of nanofluid by considering the conductivity of each material^42^:

9 $$ \begin{aligned} \frac{{k_{hnf} }}{{k_{bf} }} & = \frac{{k_{s2} + \left( {s - 1} \right)k_{bf} - \left( {s - 1} \right)\phi_{2} \left( {k_{bf} - k_{s2} } \right)}}{{k_{s2} + \left( {s - 1} \right)k_{bf} + \phi_{2} \left( {k_{bf} - k_{s2} } \right)}}, \\ \frac{{k_{bf} }}{{k_{f} }} & = \frac{{k_{s1} + \left( {s - 1} \right)k_{f} - \left( {s - 1} \right)\phi_{1} \left( {k_{f} - k_{s1} } \right)}}{{k_{s1} + \left( {s - 1} \right)k_{f} + \phi_{1} \left( {k_{f} - k_{s1} } \right)}} \\ \end{aligned} $$

Using the Brinkman model, the dynamic viscosity of a hybrid nanofluid can be calculated using the accompanying equation^42^:

10 $$\upmu _{{{\text{hnf}}}} { = }\frac{{\upmu _{{\text{f}}} }}{{(1 - \phi_{1} )^{2.5} (1 - \phi_{2} )^{2.5} }} $$

The thermophysical properties of selected nanofluids are shown in Table 1. The primary point in the dimensional analysis of a system is to determine the number of dimensionless parameters that can be substituted for the main variables. non-dimensionalization solves the complexity and reduces the number of influential variables in a physical phenomenon, which provides a clear perspective in the analysis process and is very beneficial.

**Table 1:** Details of nanoparticles utilized in the present study.

Physical properties	Water/ethylene glycol	GO	Al_2_O_3_
$\rho \,\,\,(\frac{kg}{{m^{3} }})$	1063.8	1800	3970
$c_{p} \,\,\,(\frac{J}{{kg.^{ \circ } K}})$	3630	717	765
$k\,\,\,(\frac{W}{{m.^{ \circ } K}})$	0.387	5000	25
$\beta \times 10^{ - 5} (k^{ - 1} )$	58	1.6	1.26

Thus, the stream function and dimensionless parameters is displayed below:

11 $$ \begin{aligned} & v = - \frac{\partial \psi }{{\partial x}},\quad u = \frac{\partial \psi }{{\partial y}},\quad \Delta T = \frac{{Lq^{\prime\prime}}}{{k_{f} }}, \\ & \theta = \frac{{\left( {T - T_{c} } \right)}}{{\left( {\Delta T} \right)}},\quad \left( {X,Y} \right) = \frac{{\left( {x,y} \right)}}{L},\quad \Psi = \frac{\psi }{{\alpha_{nf} }} \\ \end{aligned} $$

As a result, the ultimate form of dimensionless equations in Eq. (12) and Eq. (13) is presented. The dimensionless physical parameters are inserted into the equations, and these obtained equations are used to perform simulations.

12 $$ \begin{aligned} \frac{{\partial^{2} \Psi }}{{\partial X^{2} }} + \frac{{\partial^{2} \Psi }}{{\partial Y^{2} }} & = - \frac{{A_{6} }}{{A_{5} }}Ha\left[ {\frac{{\partial^{2} \Psi }}{{\partial Y^{2} }}\left( {\sin^{2} \gamma } \right) + \frac{{\partial^{2} \Psi }}{{\partial X^{2} }}\left( {\cos^{2} \gamma } \right) + 2\frac{{\partial^{2} \Psi }}{\partial X\partial Y}\left( {\sin \gamma } \right)\left( {\cos \gamma } \right)} \right] \\ & \quad - \frac{{A_{3} A_{2} }}{{A_{4} A_{5} }}\frac{\partial \theta }{{\partial X}}Ra\, \\ \end{aligned} $$

13 $$ \varepsilon \left( {\left( {\frac{{\partial^{2} \theta }}{{\partial X^{2} }}} \right) + \left( {1 + \frac{4}{3}\left( {\frac{{k_{f} }}{{k_{hnf} }}} \right)Rd} \right)\left( {\frac{{\partial^{2} \theta }}{{\partial Y^{2} }}} \right)} \right) = - \frac{\partial \theta }{{\partial Y}}\frac{\partial \Psi }{{\partial X}} + \frac{\partial \Psi }{{\partial Y}}\frac{\partial \theta }{{\partial X}}\, $$

The constant parameters within the above equations are characterized as follows:

14 $$ \begin{aligned} & A_{1} = \frac{{\rho_{hnf} }}{{\rho_{f} }},\quad A_{2} = \frac{{\left( {\rho C_{p} } \right)_{hnf} }}{{\left( {\rho C_{p} } \right)_{f} }},\quad A_{3} = \frac{{\left( {\rho \beta } \right)_{hnf} }}{{\left( {\rho \beta } \right)_{f} }},\quad A_{4} = \frac{{k_{hnf} }}{{k_{f} }},\quad A_{5} = \frac{{\mu_{hnf} }}{{\mu_{f} }},\quad A_{6} = \frac{{\sigma_{hnf} }}{{\sigma_{f} }}, \\ & Ra = \frac{{gK\left( {\rho \beta } \right)_{f} L\Delta T}}{{\mu_{f} \alpha_{f} }},\quad Ha = \frac{{\sigma_{f} KB_{0}^{2} }}{{\mu_{f} }},\quad \delta_{s} = \frac{{k_{hnf} }}{{\left[ {k_{s} \left( {1 - \varepsilon } \right)} \right]}} \\ \end{aligned} $$

Boundary conditions can be considered as follows:

15 $$ \begin{array}{*{20}l} {\Psi = 0.0} \hfill & {\quad \quad on\,all\,walls} \hfill \\ {\theta = 0.0} \hfill & {\quad \quad on\,outer\,wall} \hfill \\ {\theta = 1.0} \hfill & {\quad \quad on\,inner\,wall\,\,and\,outer\,wavy\,wall} \hfill \\ \end{array} $$

In order to accurately analyze the amount of natural convection in the cavity, the Nusselt number, one of the essential indicators in heat transfer, is calculated, so its local and average ratios are calculated from Eqs. (16 and 17).

16 $$ Nu_{loc} = \left( {\frac{{k_{hnf} }}{{k_{f} }}} \right)\frac{1}{\theta }\left( {1 + \frac{4}{3}\left( {\frac{{k_{f} }}{{k_{hnf} }}} \right)Rd} \right) $$

17 $$ Nu_{ave} = \frac{1}{2\pi }\int\limits_{0}^{2\pi } {Nu_{loc} dr} $$

## Numerical solution procedure

**Figure 2:**
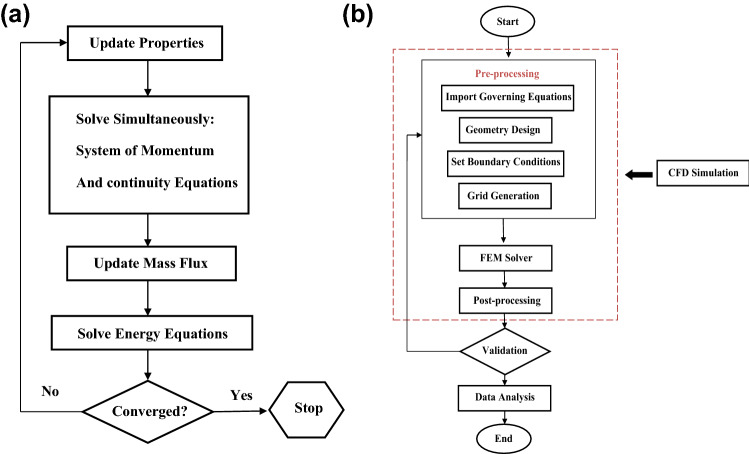
(**a**) The flowchart of the computational algorithm, (**b**) the flowchart of the numerical procedure.

The standard Galerkin FEM (GFEM) is used to solve the governing equations of the flow. This method provides reliable results due to its high strength and flexibility. The simulations of this research have been done using coding in FlexPDE software, which is an open-source software. Once the initial meshing is finished, the software continuously calculates the remaining solution. It may also change the mesh structure to achieve acceptable accuracy. The software also facilitates the creation of mesh networks by introducing a dynamic mesh domain that is continuously refined or coarsened depending on how close the solution is to convergence in neighboring cells. Moreover, the control volume-based finite element (CVFEM) method is a powerful numerical method that combines finite volume and finite element methods to discretize complex geometries with a unique formulation. Therefore, the variables are easily analyzed in terms of flux, power and resources. Figure 2 depicts the algorithm created to understand the numerical solution technique better.

## Results and discussion

### Meshing independency and validation assessment

**Figure 3:**
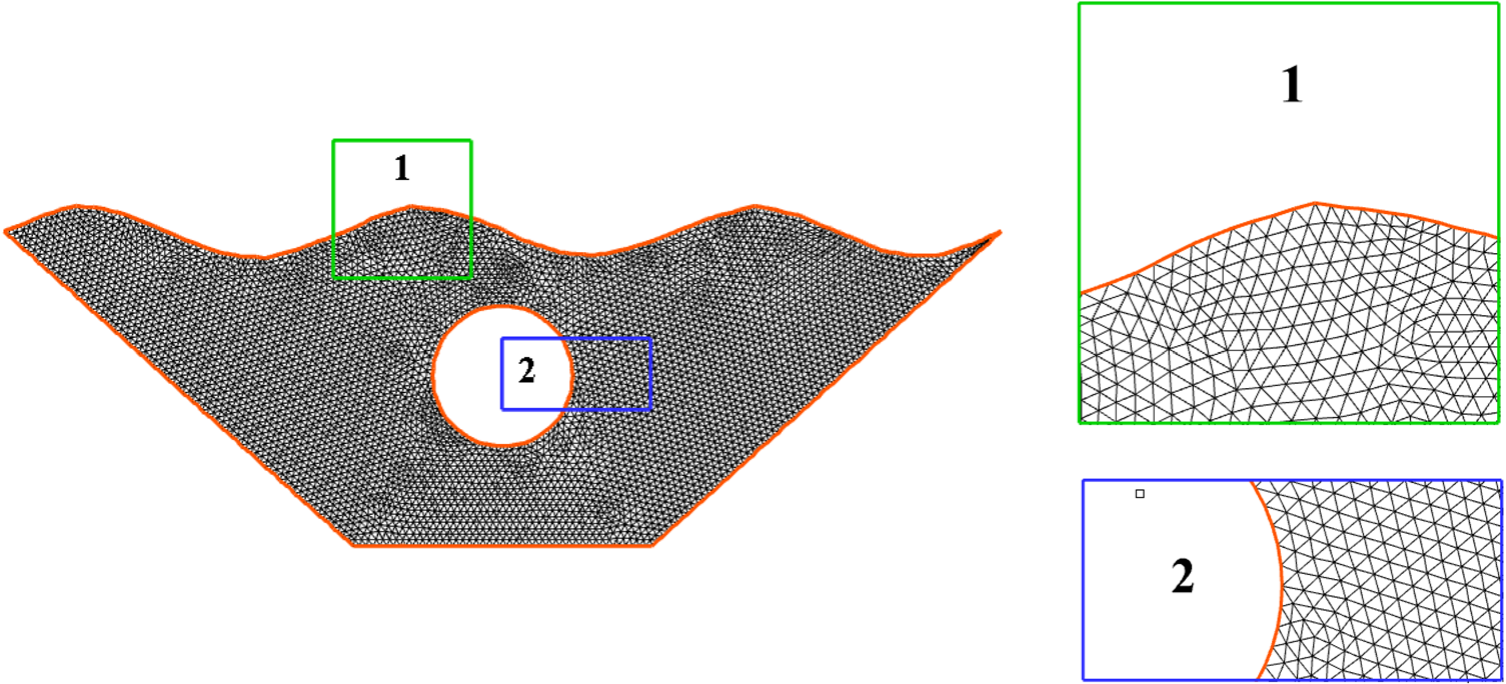
Adaptive mesh refinement.

**Figure 4:**
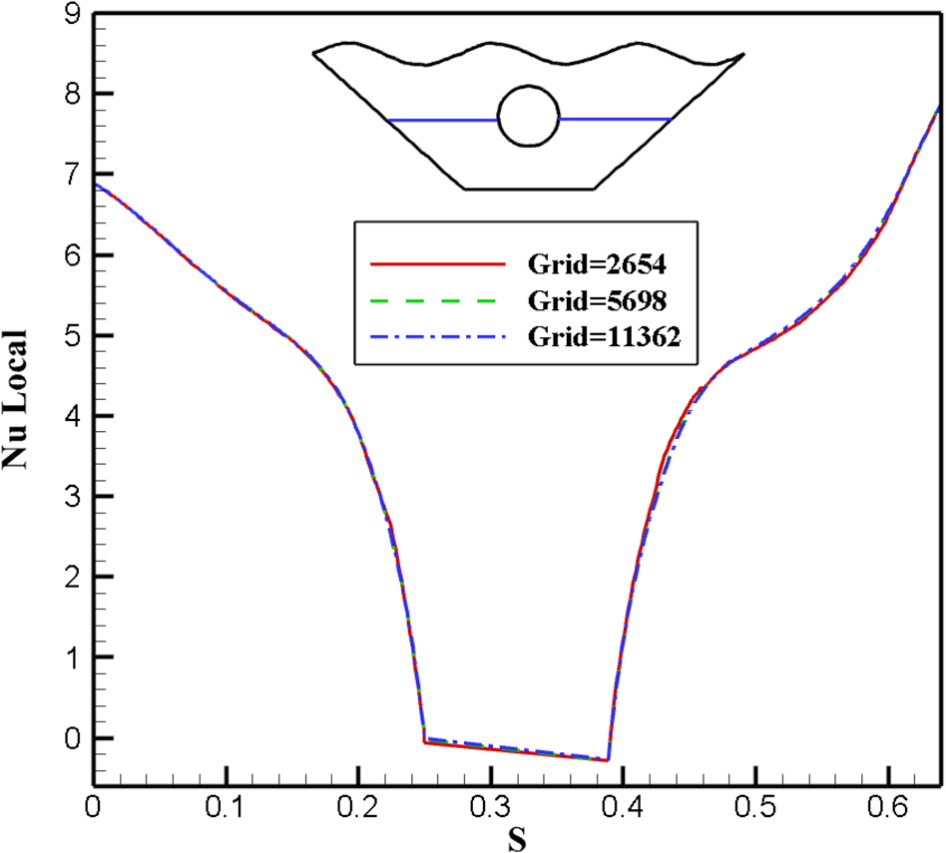
Comparison of the local Nu number for different grid sizes when $Ra = \,500,\,\,Ha\, = 20,\,\,\varepsilon \, = \,0.7,\,\,Rd = 0.5,\,\,\alpha = \pi /6$.

**Figure 5:**
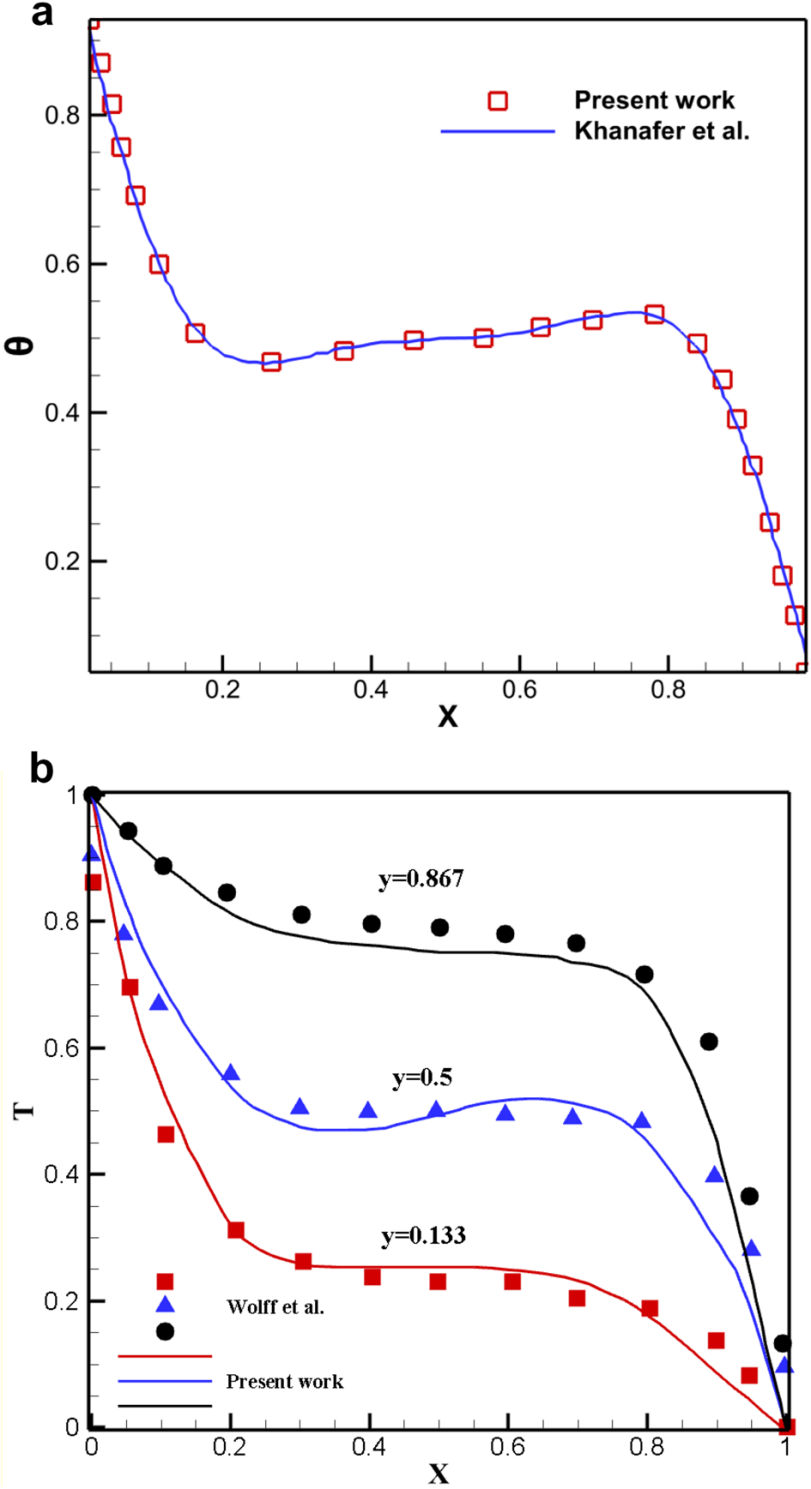
**(a)** Validation of the present study with Khanfar et al.^43^. $\phi = 0.05$ and $\Pr = 6.2$ (Cu–Water). (**b**) Validation of the present study with the experimental work of Wolff et al.^44^. Z = 0.5, $Ra = 1.689 \times 10^5$.

Any numerical analysis that attempts to reflect and resemble the real-life problem should generate a grid structure of sufficient quality so that further meshing refinement does not affect the results. Additionally, as the computational cost increases exponentially with a more sophisticated meshing framework, higher quality for the grid network frequently translates to a more laborious process. As a result, the perfect network should strike a balance between price and accuracy. Figure 3 shows this. In order to guarantee that there is a balance between precision and cost, a flexible and dynamic computational field is used. Concerning the problem's overall converging state, each section's density is entirely independent and is attributed to its converging criteria. In order to further demonstrate the validity of this dynamic network method, the local Nu at the inner cavity for three networks with various elemental densities ranging from roughly 3000 to 12,000 elements is presented in Fig. 4. The difference in Nu values can generally be detected. Because it strikes a better balance between price and accuracy and offers a quicker solution, the 5698-element grid is chosen as the computational field for study. To evaluate the precision of the numerical solution method, it is essential to compare the results of the present study with the results of previous articles. Therefore, Fig. 5a shows a comparison between this study and the results of research by Khanafer et al.^43^, it is clear that the results of both studies are in line with each other and are well-matched, so it can be concluded that the outputs obtained from the method under discussion are accurate and stable. In addition, the experimental findings of Wolff et al.^44^ has been compared for natural convection in a cubic cavity with different temperatures as the second validity of the present study. The comparison depicted that the temperature profiles in various locations are in excellent agreement, as shown in Fig. 5b.

### Numerical analysis

This study describes the effects of natural convection, porous medium porosity, Rayleigh dimensionless parameter, radiation strength, and effectiveness of magnetic fields on a nanofluid flow field under isothermal boundary conditions for a wavy trapezoidal porous cavity containing Mixture Hybrid nanofluid. The upper wall of the cavity was analyzed for different wave numbers (N = 0, 1, 2, 3, 4) using the highest local Nusselt and the best option, N = 3, was selected to perform the simulations. For each determinant, the resulting figures are presented with three different scales streamlined contours, isotherm profiles, and local Nu plots. The set points for determining porosity are 0.5, 0.7, and 0.9, while the Rayleigh number scale alternates between 100, 500, and 1500. The Ha number, which indicates the strength of the magnetic field, ranges from zero, which indicates that there is no external field, to 20 and 40, respectively, in this analysis. To better understand its impact, the radiation factor is changed from 0 to 0.5 and then to 1.

#### The number of waves upper wall effect (N)

**Figure 6:**
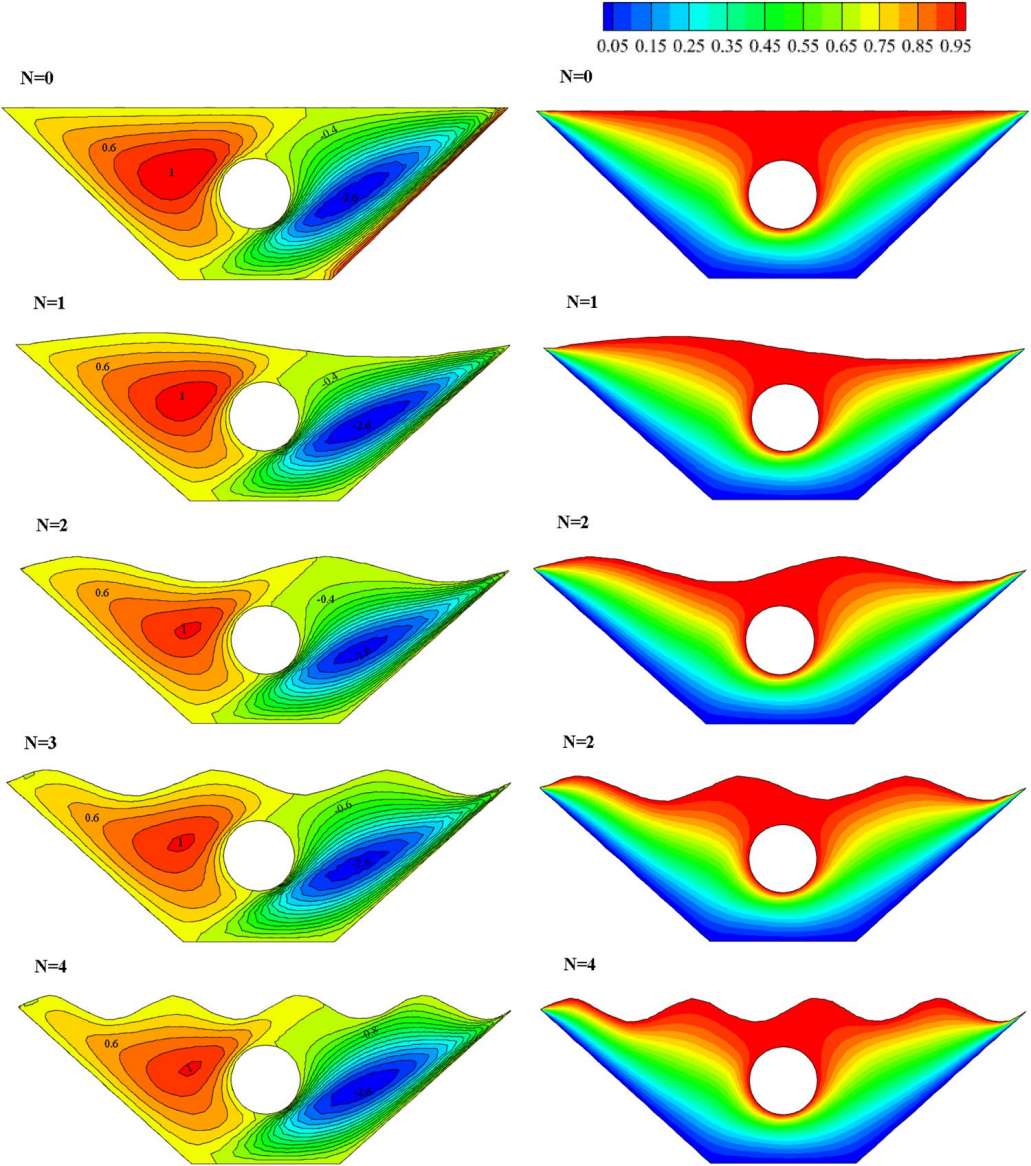
Stream functions and Isotherms for different number of waves (N) at ℇ = 0.7, Ra = 500, Ha = 20, Rd = 0.5 and α = π/6.

**Figure 7:**
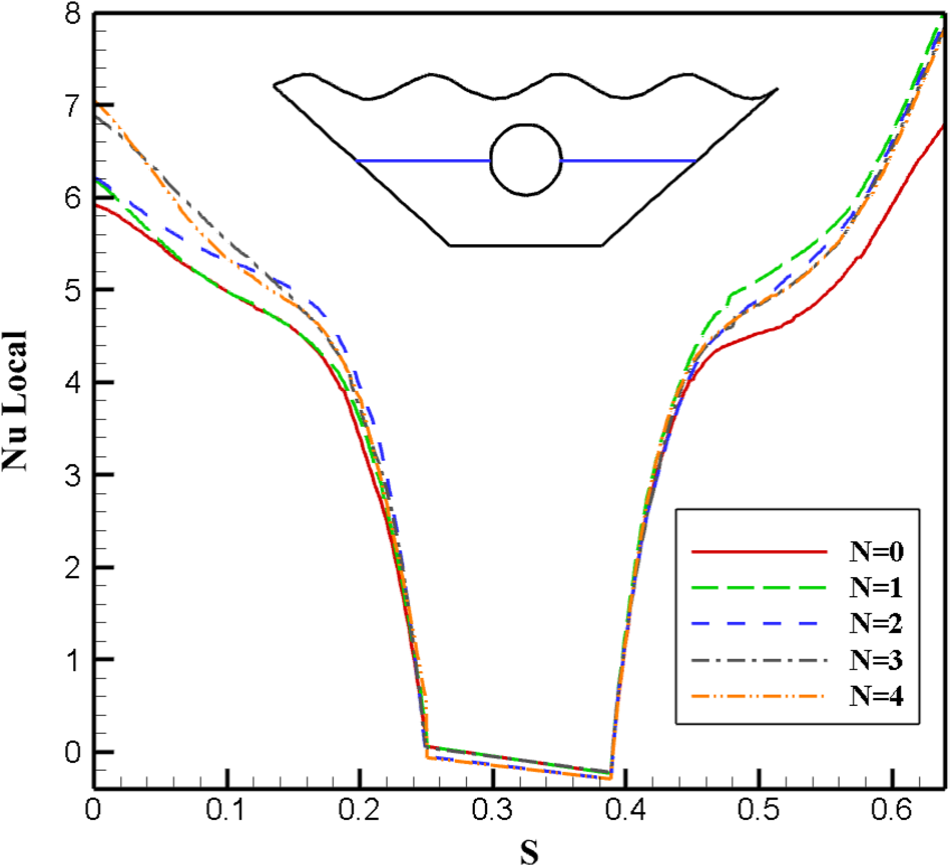
Local Nu number plot for different number of waves (N).

Stream functions and isotherms for a different number of upper wall waves (N = 0, 1, 2, 3, 4) are given in Fig. 6, considering average values for porosity, Rayleigh number, radiation, Hartmann number and magnetic field angle. Inside the chamber, two vortices are created in opposite directions. The vortex on the right rotates clockwise, while the vortex on the left is positive and rotates counterclockwise. In the vicinity of the inclined and lower walls, a cold fluid flows. As it gets closer to the top of the chamber, fluid temperature and heat distribution increase around the hot circular cavity and the upper corrugated wall is observed. By examining the flow lines, it is clear that with the increase in the number of waves, the shape of the flow lines changes and spreads throughout the chamber, and the best heat transfer and temperature distribution occurs at N = 3. Also, to choose the most appropriate geometry according to the number of waves, as seen in Fig. 7, the local Nu number for the constant characteristic length on the desired surface is examined in terms of the number of waves. Since more significant Nusselt numbers indicate higher convective heat transfer, from the obtained results, the highest local Nu number corresponds to the geometry with N = 3. Therefore, the desired geometry is used as the basis of analysis to apply other parameters.

#### Qualification of porosity alteration effectivity (ℇ)

**Figure 8:**
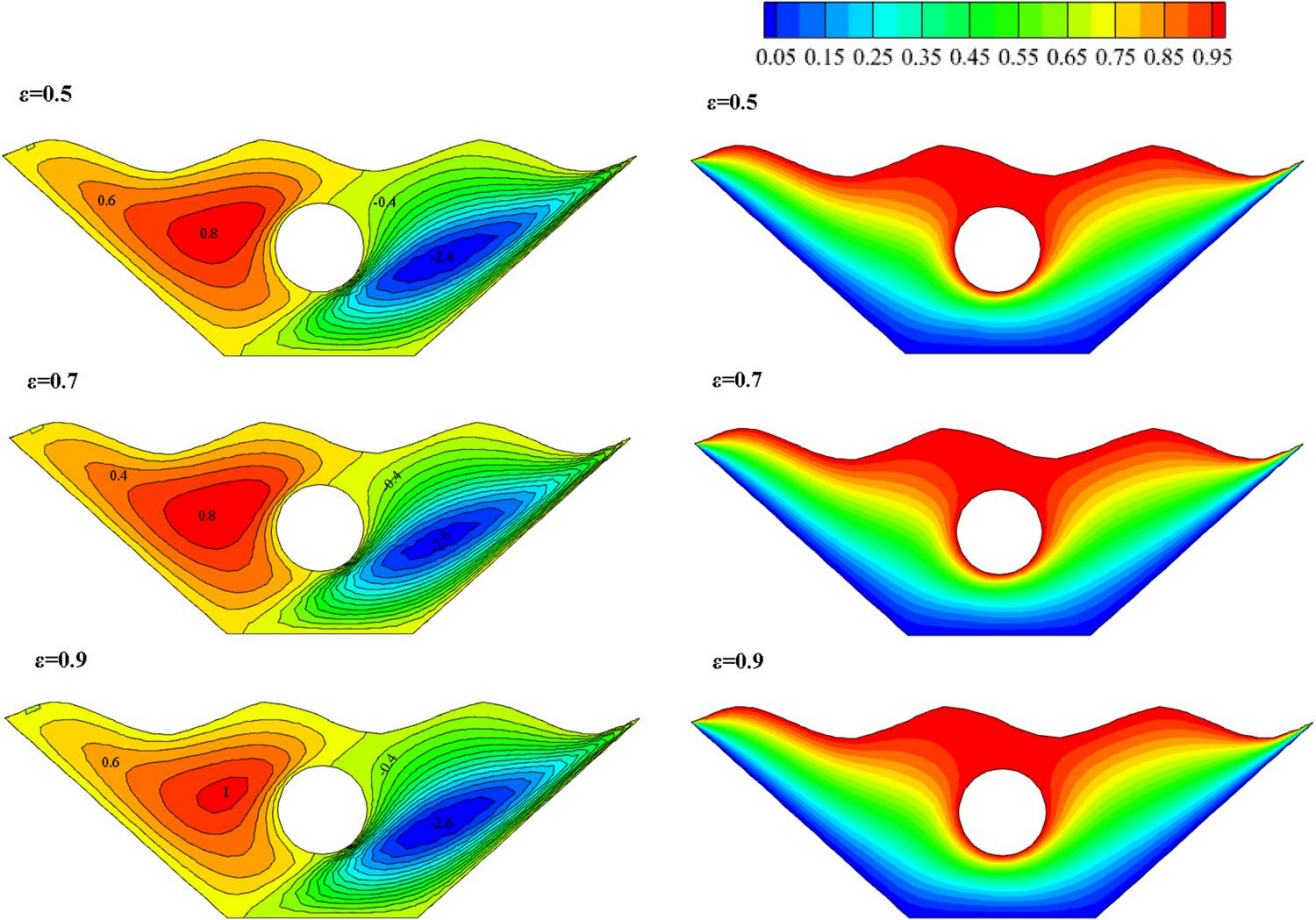
Stream functions and Isotherms for different values of porosity (ℇ) at Ra = 500, Ha = 20, Rd = 0.5 and α = π/6.

**Figure 9:**
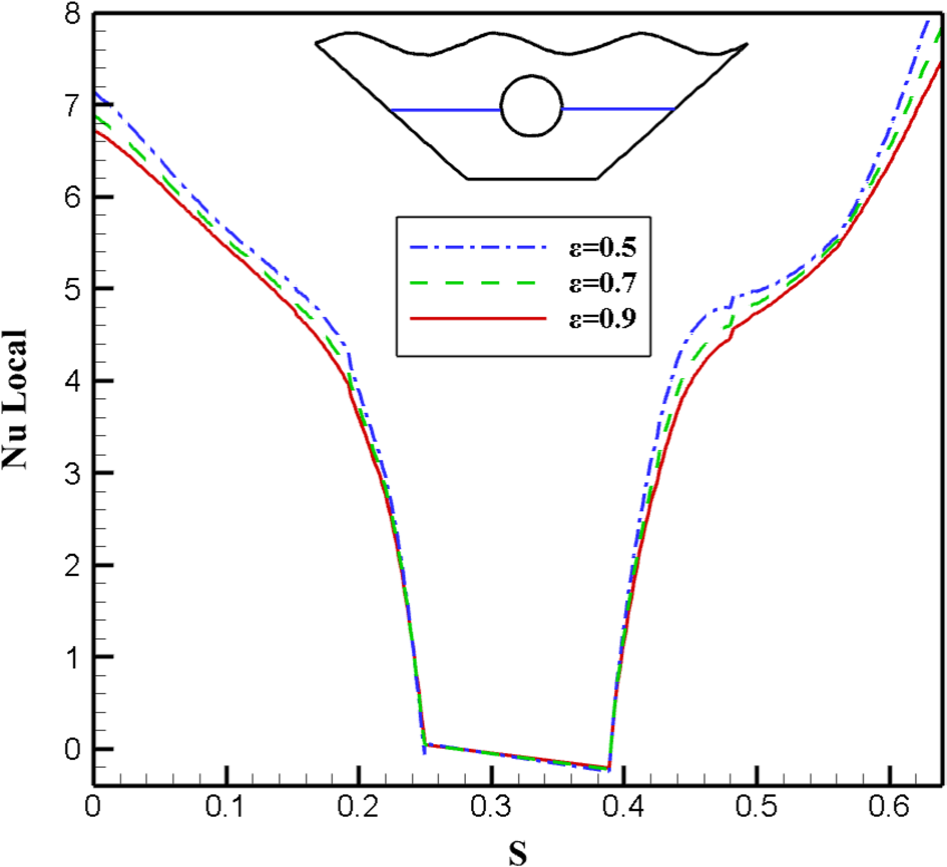
Local Nu number plot for different porosity (ℇ).

**Figure 10:**
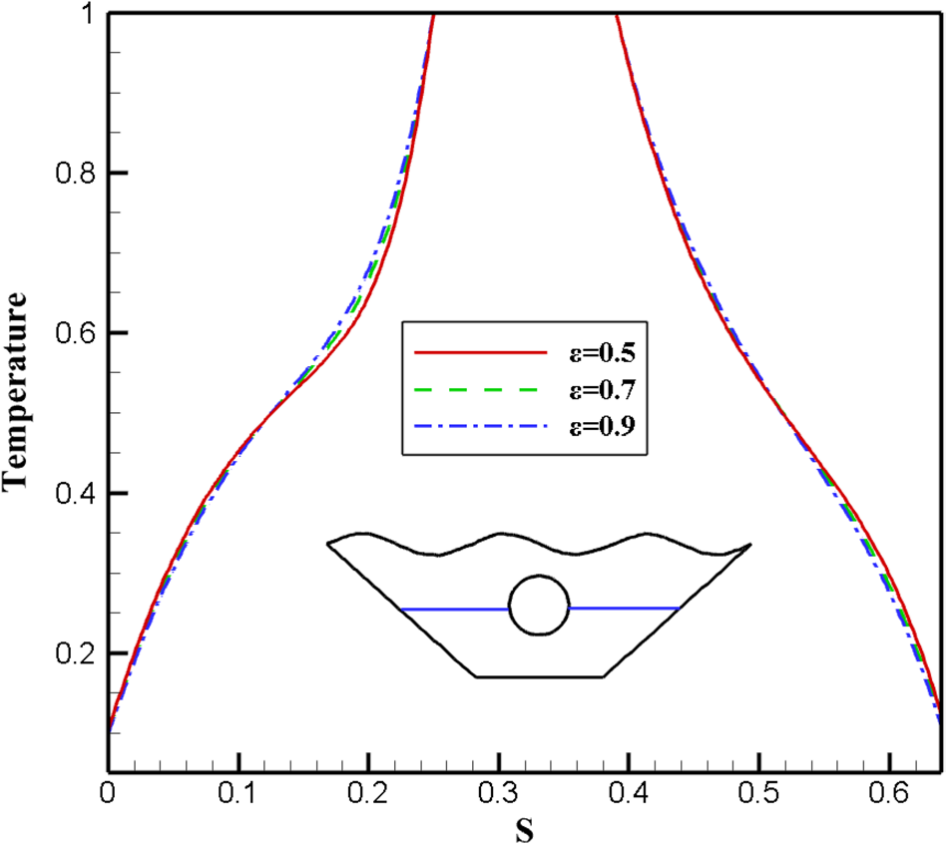
Temperature plot for different porosity (ℇ).

The effect of changes in the fluid porosity ratio inside the chamber is shown in Fig. 8. Streamlines and isotherms are obtained for porosities of 0.5, 0.7, and 0.9, keeping other effective variables constant in the mean value. As can be seen in isotherms, at a porosity of 0.5, more efficient heat transfer takes place from the hot upper wall to the lower chamber and around the inner hot cavity. To better understand this issue, the local Nusselt is investigated for three different porosity values in the direction of the specified characteristic length. Figure 9 shows that the maximum local Nusselt number obtained for porosity is 0.5, so the fluid with the lowest porosity has a higher convective than other values because the higher the Nusselt numbers, the higher the convective heat transfer rate. Figure 10 appear the effect of temperature profiles along with the specified characteristic for various porosity values. As can be seen, the 0.7 and 0.9 porosity fluid diagrams overlap more than the 0.5 porosity diagrams, and at higher temperatures, close to 1, all three diagrams overlap. With a general look at the results of the study of different porosities in the chamber, it can be seen that in a fluid with less porosity, the convective heat transfer rate is higher, and the fluid flow is stronger than other values of porosity.

#### Qualification of Rayleigh number alteration effectivity (Ra)

**Figure 11:**
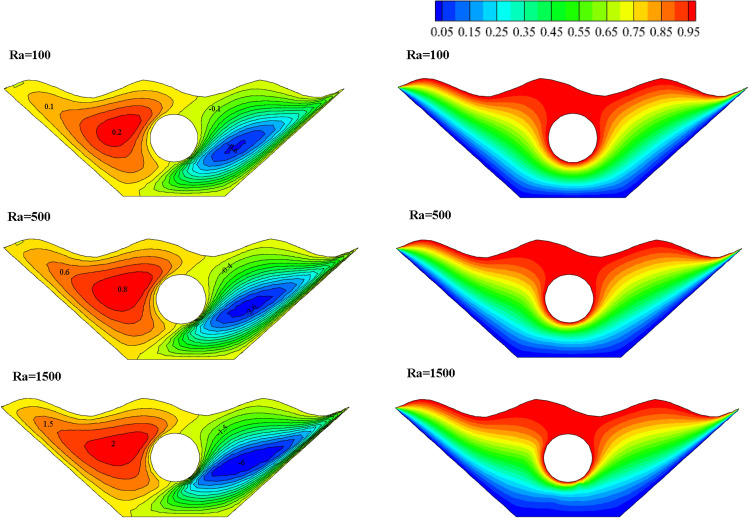
Stream functions and Isotherms for different values of Rayleigh numbers (Ra) at ℇ = 0.7, Ha = 20, Rd = 0.5 and α = π/6.

**Figure 12:**
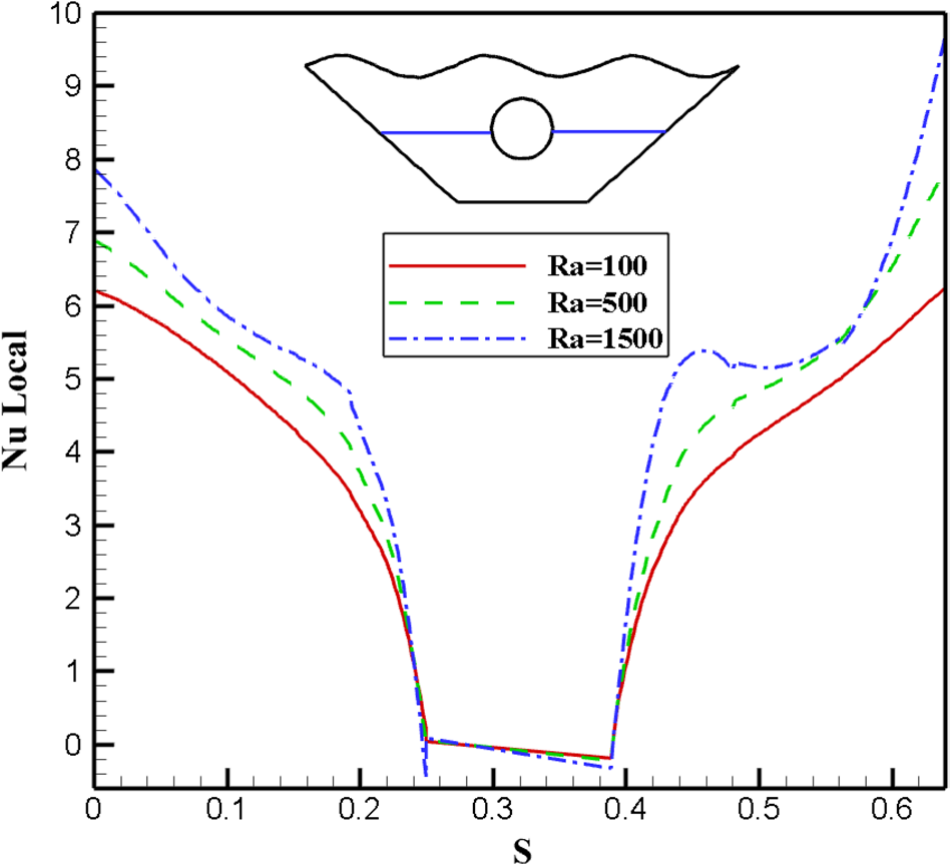
Local Nu number plot for different Ra numbers (Ra).

**Figure 13:**
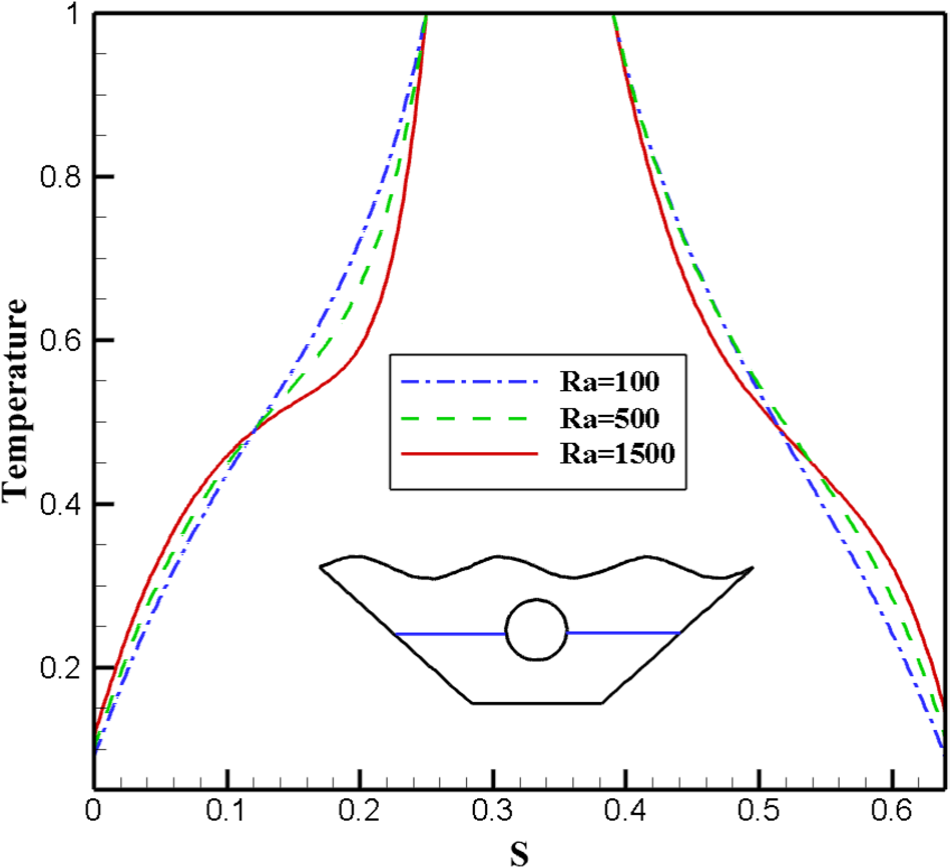
Temperature plot for different Ra numbers.

Since the Rayleigh number has three different values in this study, it has been investigated as one of the essential parameters in free convection. Figure 11 shows how the isothermal and stream lines work for it. Isotherm plots show how isotherm lines become more regular and less curved at smaller Rayleigh numbers. This shows that there is more convective heat transfer than conductive heat transfer. The ratio of buoyancy to heat loss is represented by the Rayleigh number. So at low Rayleigh numbers, flow is low due to buoyancy and the fluid transfers heat due to higher conductivity. Fluid flow and molecular movement increase with increasing Ra number. The streamlines in Fig. 11 show that with the increase of the Ra number, the value of the stream function in the center of the vortices increases dramatically. At Ra = 1500, the stream function reaches its maximum value. Due to the increase in the buoyancy force, the strength of the vortex and the speed of the flow in the chamber increases in these conditions. Three different Rayleigh number values are shown in Fig. 12 along with the local Nusselt value corresponding to the selected surface. As can be seen, the graph with Ra = 1500 has the highest Nu number and also has more curvature than the other two graphs, which indicates the improvement of heat transfer and stronger fluid flow. In Fig. 13, the effect of temperature on the Rayleigh numbers for the three values considered for further investigation is investigated. However, the plot for Ra = 1500 is very different from the results for Ra = 100 and Ra = 500. As can be seen, as the temperature increases, the curvature of the graph increases and intersects the other graphs at two points, which indicates that with the increase of temperature and the increase of Rayleigh number, there is more disorder and thus more efficient heat transfer. comes. So, looking at these graphs, the claims mentioned above are supported.

#### Qualification of Hartmann number alteration effectivity (Ha)

**Figure 14:**
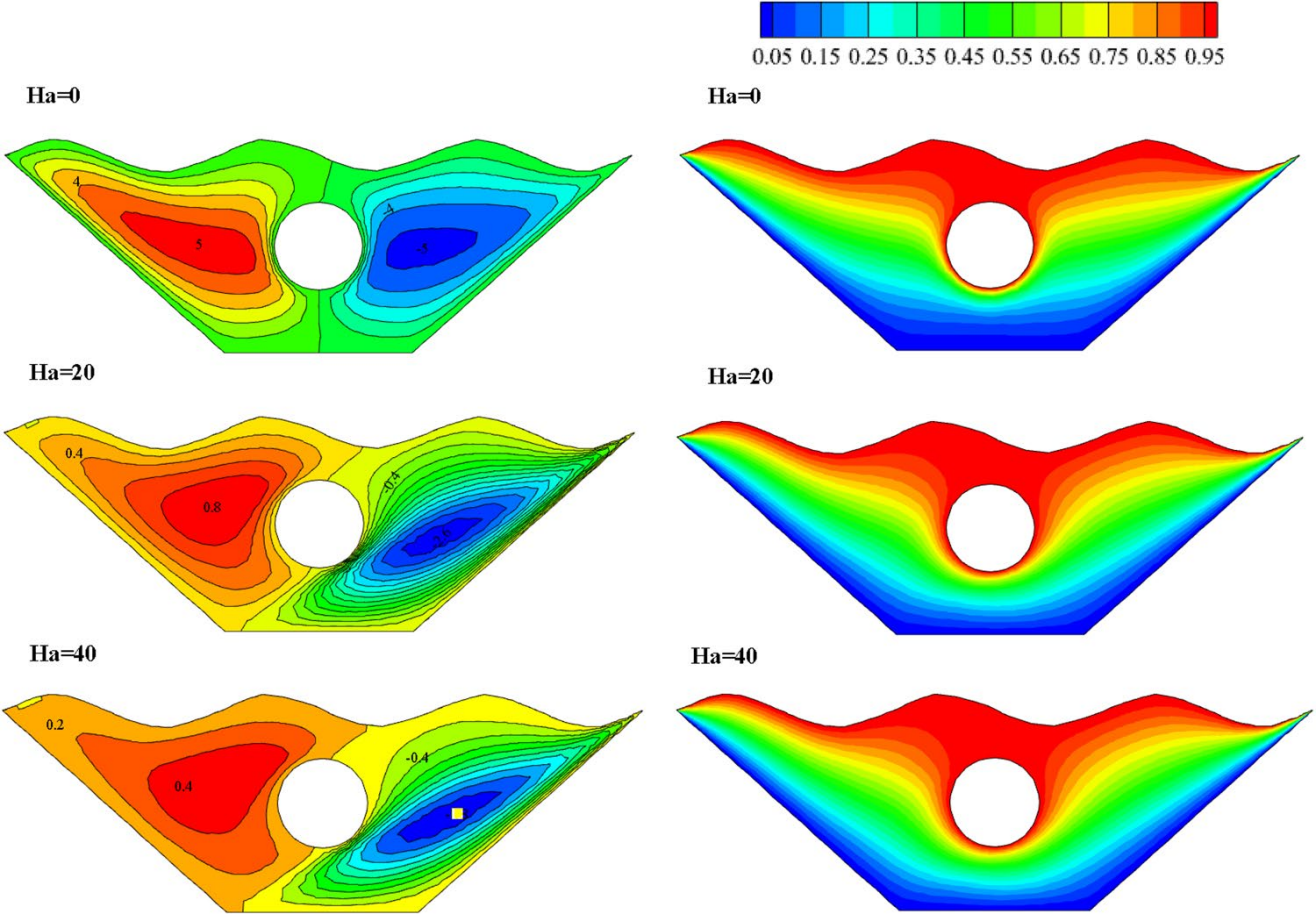
Stream functions and Isotherms for different values of Hartman numbers (Ha) at ℇ = 0.7, Ra = 500, Rd = 0.5 and α = π/6.

**Figure 15:**
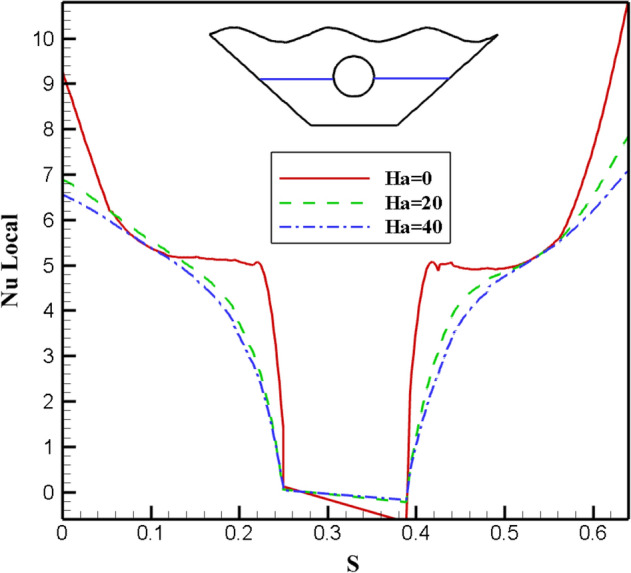
Local Nu number plot for differentHartman numbers (Ha).

**Figure 16:**
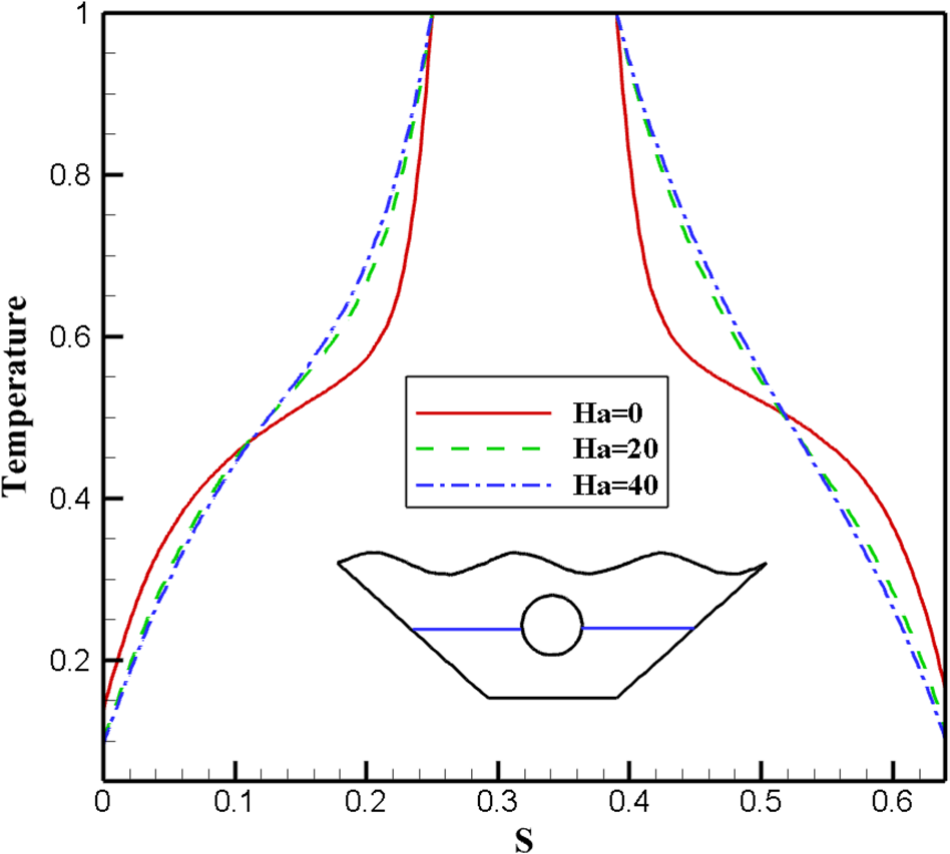
Temperature plot for various Hartman numbers (Ha).

The effects of different Ha numbers, which represent the strength of the magnetic field, are shown in Fig. 14. The effect of Ha = 0, 20, and 40 on the nanofluid inside the cavity has been studied because increasing the Ha number will result in a stronger magnetic field. A closer look at the streamlines reveals that as the Ha number rises, the vortices become weaker and are drawn toward the chamber's side walls due to the Lorentz force created by applying a magnetic field. The Lorentz force that results as the magnetic field's strength rises slows the motion of the fluid masses. Additionally, as the fluid displacement declines, the buoyancy also does so, causing slower vortices and diminished power, as in the case of the Ha = 40. As a result, Ha = 0 is where the stream function's most extreme value is in Fig. 14. It is evident in isothermal profiles that in the absence of a magnetic field, heat transfer from the corrugated surface and the hot cavity inside the chamber to the fluid inside the chamber is more effective, indicating a more significant proportion of convective heat transfer than conductive heat transfer. The highest Nusselt number was obtained for Ha = 0, which is very different from other values applied to the magnetic field. Figure 15 illustrates the impact of graphs of various values of the Ha number on local Nusselt. This outcome may support these findings. Figure 16 depicts how the Hartman number affects the temperature at various values. The graphs of Ha = 20 and Ha = 40 are obtained with a slight curvature and produce similar results. Nevertheless, the graph's curvature is significantly increased without a magnetic field, intersecting the other graphs at two points. The absence of a magnetic field results in a temperature diagram with a large curvature, which denotes a more effective convective process.

#### Qualification of Radiation factor alteration effectivity (Rd)

**Figure 17:**
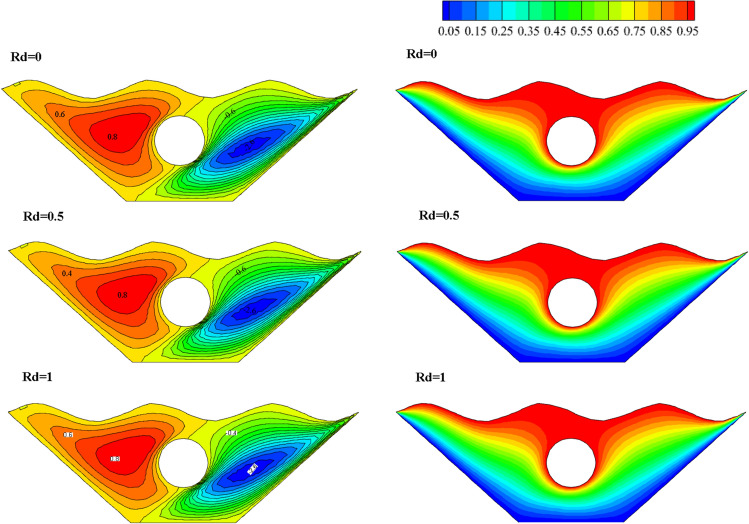
Stream functions and Isotherms for different values of Radiation (Rd) at ℇ = 0.7, Ra = 500, Ha = 20 and α = π/6.

A wavy porous enclosure makes it easier to assess Radiation when it is included in the governing equations. Rd is used as a standard for characterizing this mode of heat transfer phenomenon because it is a dimensionless form of radiation intensity. Due to the importance of the radiation parameter in heat transfer, its effect in three different values with constant values for other effective parameters has been investigated in this study. The resulting isothermal lines and streamlines are shown in Fig. 17. The reason for these ponders to increase the heat transfer rate from the heat absorbed by the corrugated surface of the chamber and the inner circular hot cavity to the fluid inside the chamber. However, by examining the results, it is observed that the streamlines for all three parameter values of Radiation provide similar results.

**Figure 18:**
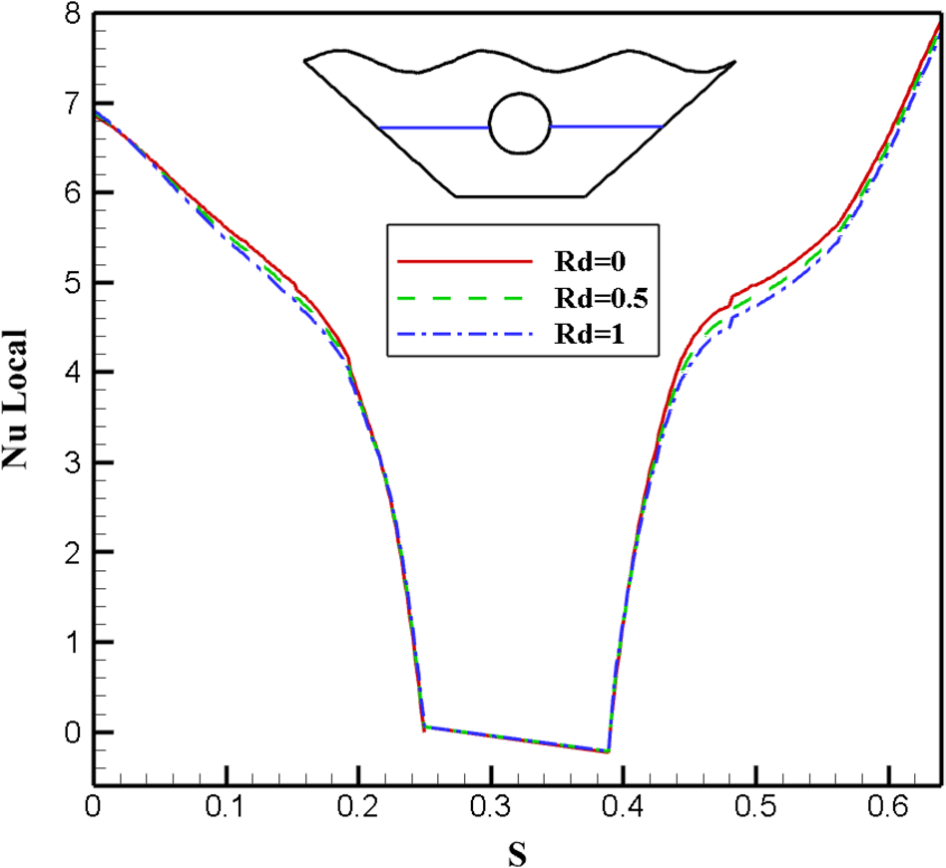
Local Nu number plot for different Radiation (Ra).

**Figure 19:**
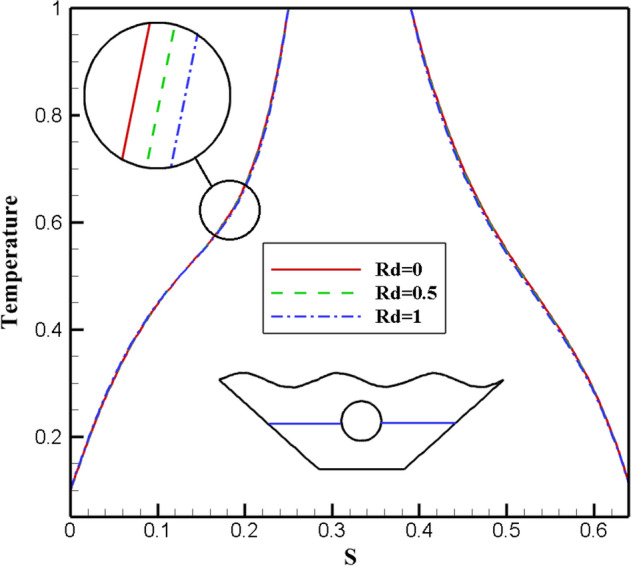
Temperature plot for different Radiation (Rd).

Similarly, in the case of isothermal lines, since the Rd is related to the heat transfer of Radiation, there is not much effect on the isotherms. For better investigation, different amounts of Radiation are shown on the local Nusselt number along the characteristic specified in Fig. 18. The results show that no significant effect is observed with increasing the amount of Radiation on the obtained Nusselt number. Figure 19 also examines the effect of Radiation on temperature, and all three graphs show similar results and are almost identical. Therefore, in general, different amounts of Radiation do not significantly affect the rate of fluid flow and heat transfer within the chamber in this numerical study.

#### Angle of magnetic field effectivity (α)

**Figure 20:**
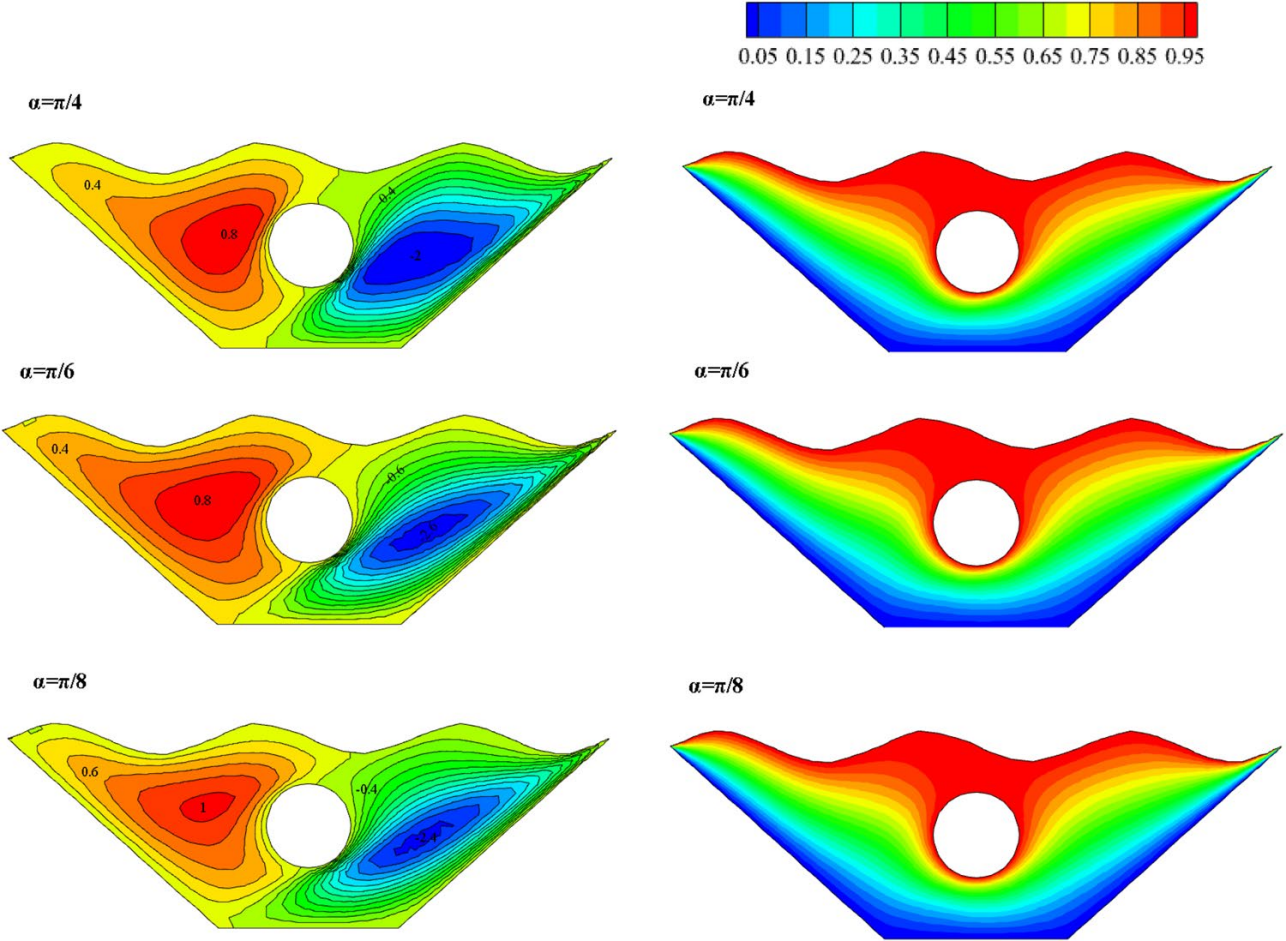
Stream functions and Isotherms for differentangle of magnetic field (α) at ℇ = 0.7, Ra = 500, Ha = 20 and Rd = 0.5

**Figure 21:**
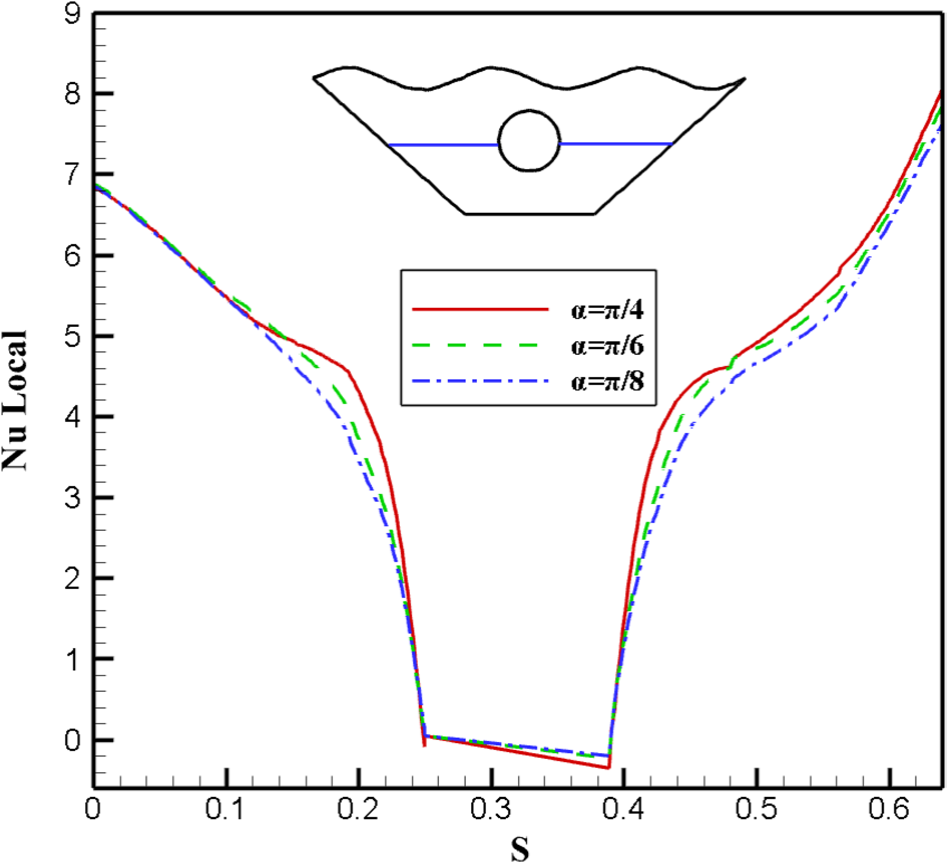
Local Nusselt number plot for differentangle of magnetic field (α).

**Figure 22:**
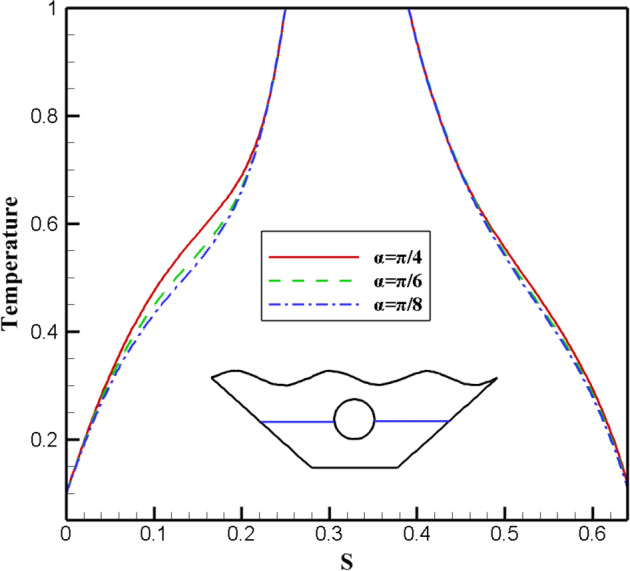
Temperature plot for different angle of magnetic field (α).

This section applies the effects of three different angle of the magnetic field to the desired geometry. Figure 20 displays the fluid inside the cavity, isotherms, and streamlined results. Applying a magnetic field with α = π/4 causes stronger and larger vortices to form on both sides, indicating an improvement in the desired hybrid nanofluid's stream functions in terms of velocity and strength. Less convective heat transfer occurs compared to conductive heat transfer as the alpha angle decreases because of the vortices' size and velocity decrease. The impact of variations on the local Nu number is examined in Fig. 21. The results show that the alpha angle (π/4) has the highest local Nusselt, indicating that the maximum heat transfer occurs at this angle. Figure 22 also depicts how it affects temperature. The graph of α = π/4 differs slightly from that of other values. The density of the isothermal lines decreases, as seen from the isothermal lines, and as temperature gradient decreases, so does the heat transfer rate within the chamber. Therefore, one of the essential variables in heat transfer within cavities is the angle of the magnetic field, which may be helpful in future engineering designs, although in some sources^10^, the distribution of nanofluid inside the chamber and the amount of heat transfer are not affected by changes in the magnetic field angle.

### Optimization and correlation development

A better analysis that can succinctly illustrate the efficacy of each additional variable, both individually and collectively, is required due to a large number of significant parameters in this problem. This study investigated five parameters: the radiation factor, the porosity, the magnetic field, angle of magnetic field and the strength of natural convection using nondimensionalized numbers at three specifications. In order to cover a fully crossed design, 81 trials would typically be needed, which is a burdensome number. This is avoided by the Taguchi method, which also offers a more detailed view of the problem at hand while introducing an alternative solution to reduce the number of trials significantly. Even though its original purpose was to allow for higher quality goods and products, this is why it has gained significant applicability in many disciplines and contexts. The system, parameter, and tolerance designs are the three essential steps this method requires to fully achieve satisfying and economical procedures. Following these guidelines reduced the number of trials from 81 to just 16 runs, significantly reducing the computational cost and onerous postprocessing. Table 2 provides the specific data for trials that were ultimately designed.

**Table 2:** Planned tests by Taguchi strategy.

Case Number	Rayleigh number	Hartman number	Porosity parameter	Radiation	$\alpha$
1	500	0	0.65	0.7	60
2	1500	0	0.9	0.35	45
3	500	30	0.9	0	30
4	100	30	0.8	0.7	45
5	1500	30	0.65	1	22.5
6	100	0	0.5	0	22.5
7	100	40	0.9	1	60
8	1000	30	0.5	0.35	60
9	500	40	0.8	0.35	22.5
10	1000	40	0.65	0	45
11	100	15	0.65	0.35	30
12	1500	40	0.5	0.7	30
13	500	15	0.5	1	45
14	1000	0	0.8	1	30
15	1000	15	0.9	0.7	22.5
16	1500	15	0.8	0	60

Response surface methodology, or RSM, has been extensively used in various optimization issues due to its simple mathematical foundations and high output accuracy. The final correlations for the response variables eliminate the need for time-consuming numerical trials and provide a more refined perspective on how each determinant interacts with the others. The use of RSM may be more advantageous because the problem's taken-into-account parameters span continuous zones. The average Nusselt number and five dimensionless control factors, Ha, Rd, Ra, ℇ, α and, make up the response variable for the anticipated correlation from RSM integration with GFEM. Compared to the numerical output dealt with in the next section, the rounding, divergence, and iteration figures may produce some potential errors. A fundamental component of RSM development is obtaining a correlation that gradually becomes more in line with the data. Therefore, polynomial expressions take precedence due to their direct interaction and adaptability. In cases of the preliminary agreement, the order of polynomials tends to increase to consider more complex issues. A typical second-order polynomial is defined as follows.

18 $$ y = a_{0} + \mathop \sum \limits_{i = 1}^{n} a_{i} x_{i} + \mathop \sum \limits_{i = 1}^{n} a_{ii} x_{i}^{2} + \mathop \sum \limits_{i = 1}^{n} \mathop \sum \limits_{j = 1}^{n} a_{ij} x_{i} x_{j} |_{i < j} + \xi $$

In this relation, Xj and Xi are the design variables, the coefficients are denoted by α, and the number of variables and errors is specific by ζ.

**Figure 23:**
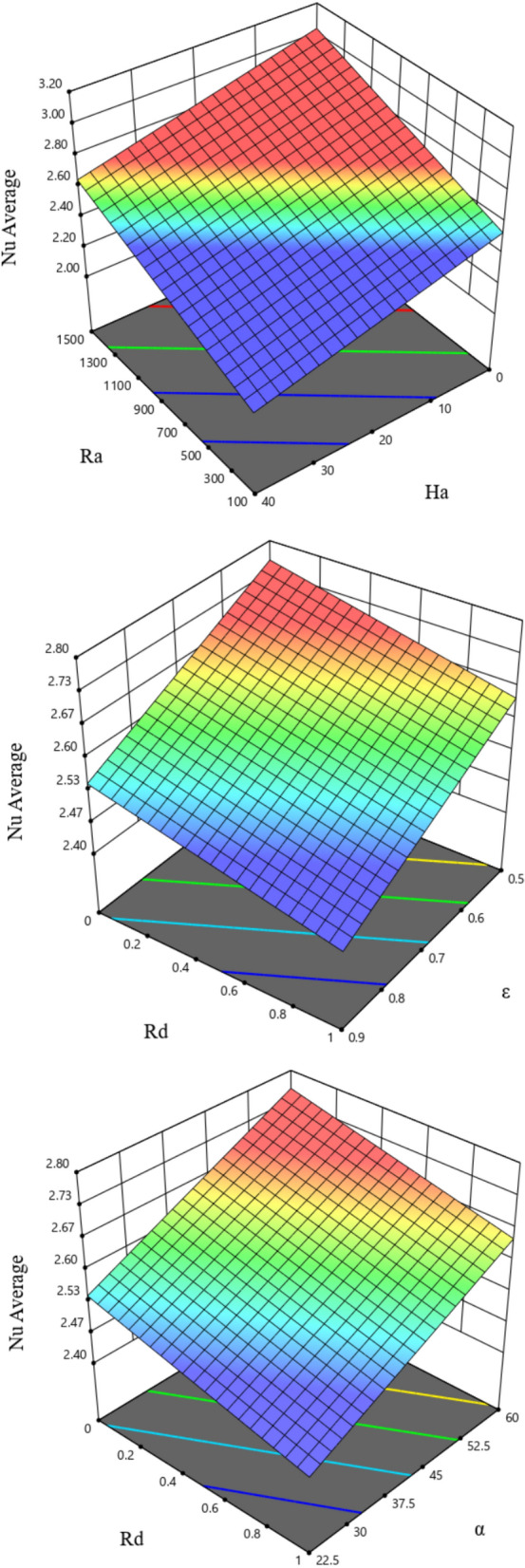
Impacts of different working parameters on average Nu number by RSM.

Figure 23 shows the three-dimensional graphs of the Nu_avg_ analysis with two pairs of parameters interactively from the RSM method. First, the interactive effect of Ha and Ra on the Nuavg is shown as the Ra number increases and the Nu_avg_ value increases. In contrast, at higher Hartmann numbers, the Nu_avg_ decreases. The following interactive effect is the porosity and radiation factor. According to the diagram, reducing the amount of porosity and the amount of radiation factor leads to a higher Nu_avg_. In the last graph, the interactive effect of α and Rd can be seen, which increases the α and, on the other hand, decreases the radiation, resulting in a higher Nu number. With the help of the desirability function approach, an estimated response is converted into a scale-free value called desirability. Optimization goals can be maximizing, minimizing, or achieving the desired response value. Between 0 and 1 is the ideal range. An unacceptable configuration for the chosen response is represented by a 0 or elementary desirability, taking the value zero.

**Figure 24:**
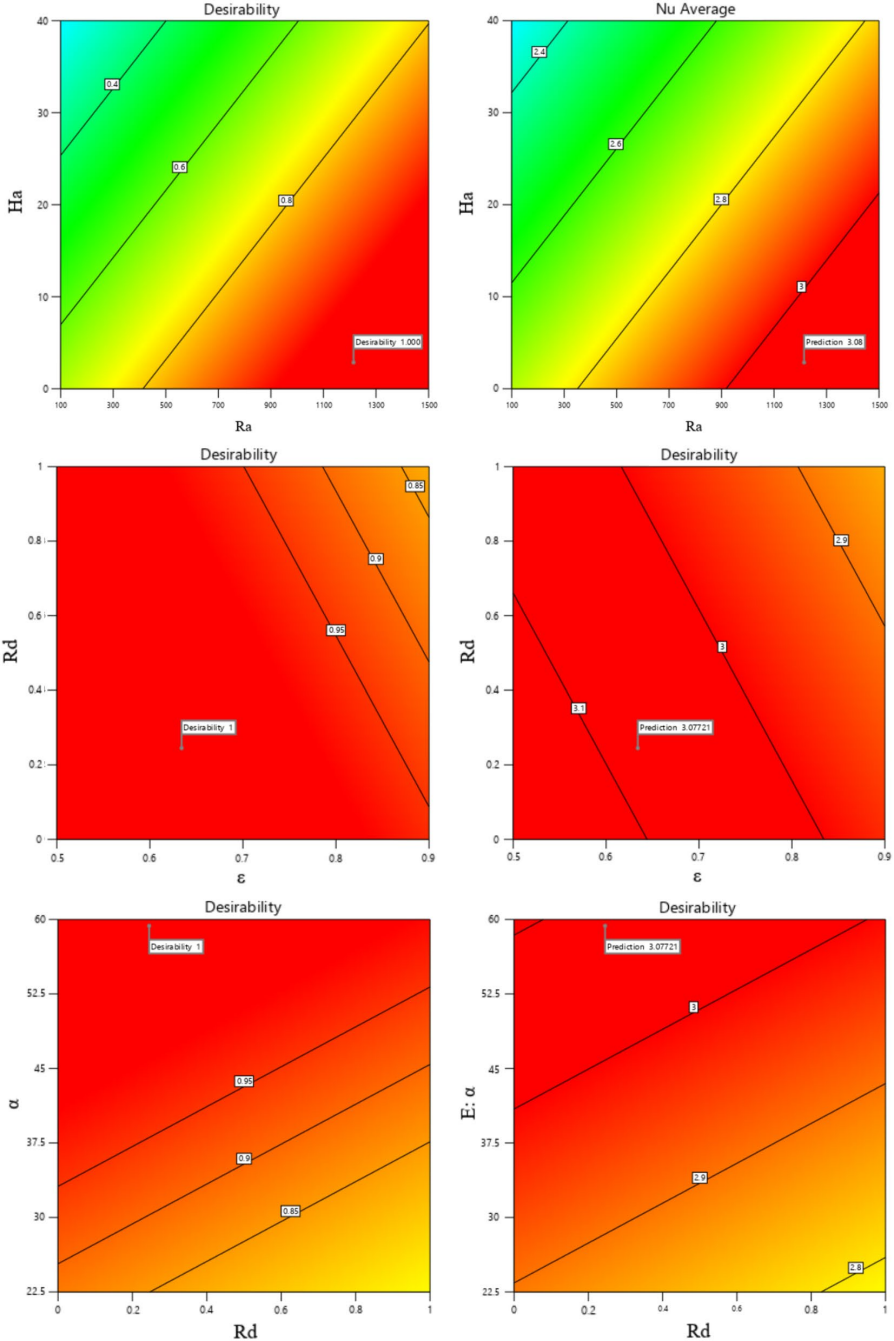
Desirability diagram of different working parameters on average Nu number by RSM.

**Figure 25:**
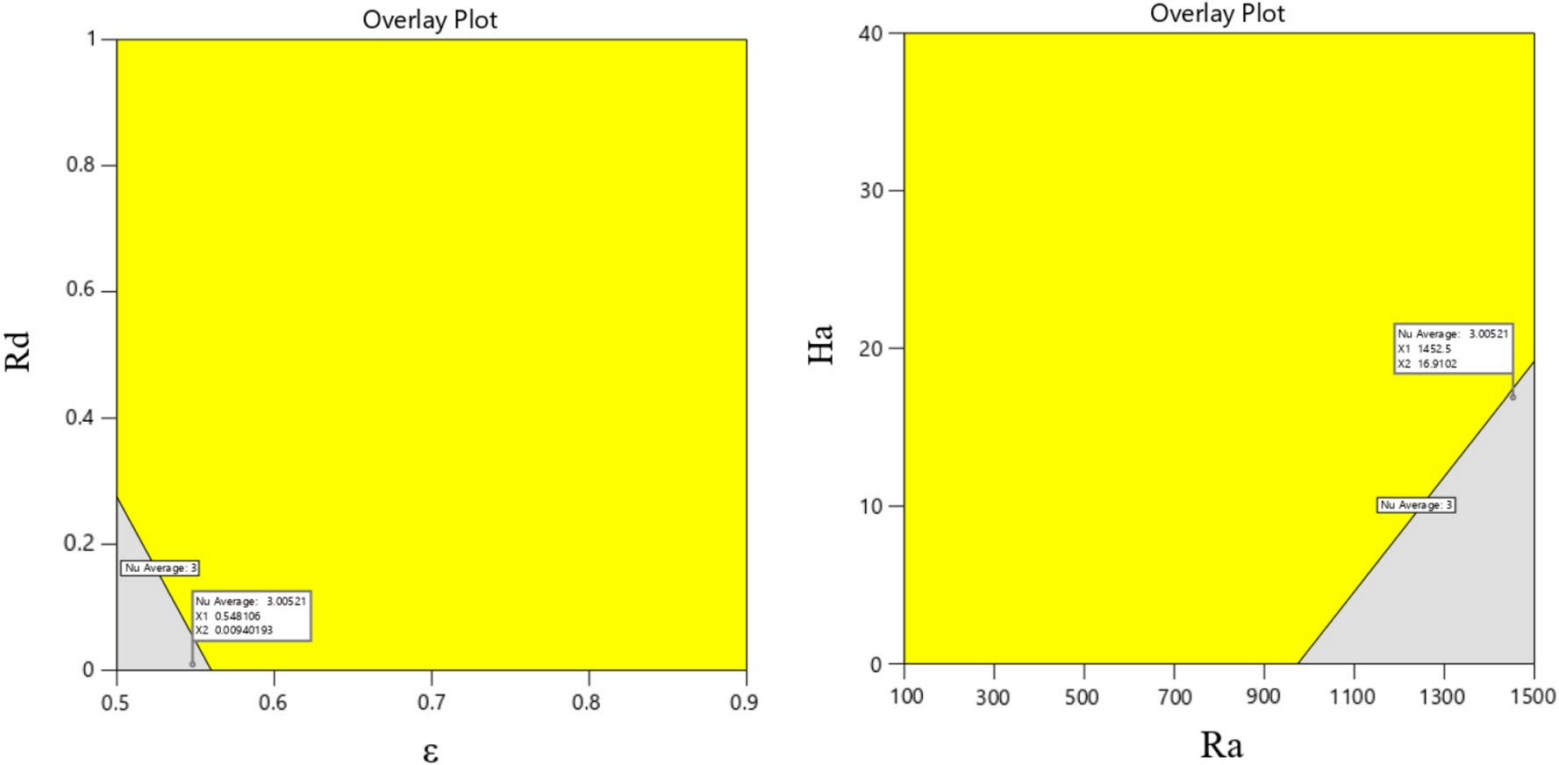
Overlay plots.

In contrast, the ideal case is illustrated by desirability taking the value 1. Figure 24 shows the desirability graphs influenced by two-parameter interactive factors for predicting the average Nusselt value. Based on this, the average Nusselt values according to the desirability of 1 in each pair of interactive parameters are displayed on the figure by flags. To show the uncertainty in the point predictions, interval estimates can be applied to the optimization graphic. Interval estimates can also be used to understand how uncertainty affects achieving process goals. The bright yellow zone in Fig. 25 denotes that every range of intervals satisfies the required standards. On the other hand, the dark gold zone refers to the region where a piece of an interval estimate does not meet the criterion requirements but the point estimate does. Equation 19 is presented to obtain the average Nu, a comprehensive relationship according to the dimensionless variables investigated in this study.

19 $$ Nu_{average} = 2.69 - 0.5255\left( \varepsilon \right) - 0.0096\left( {Ha} \right) + 0.00035\left( {Ra} \right) - 0.114\left( {Rd} \right) + 0.00571(\alpha ) $$

**Figure 26:**
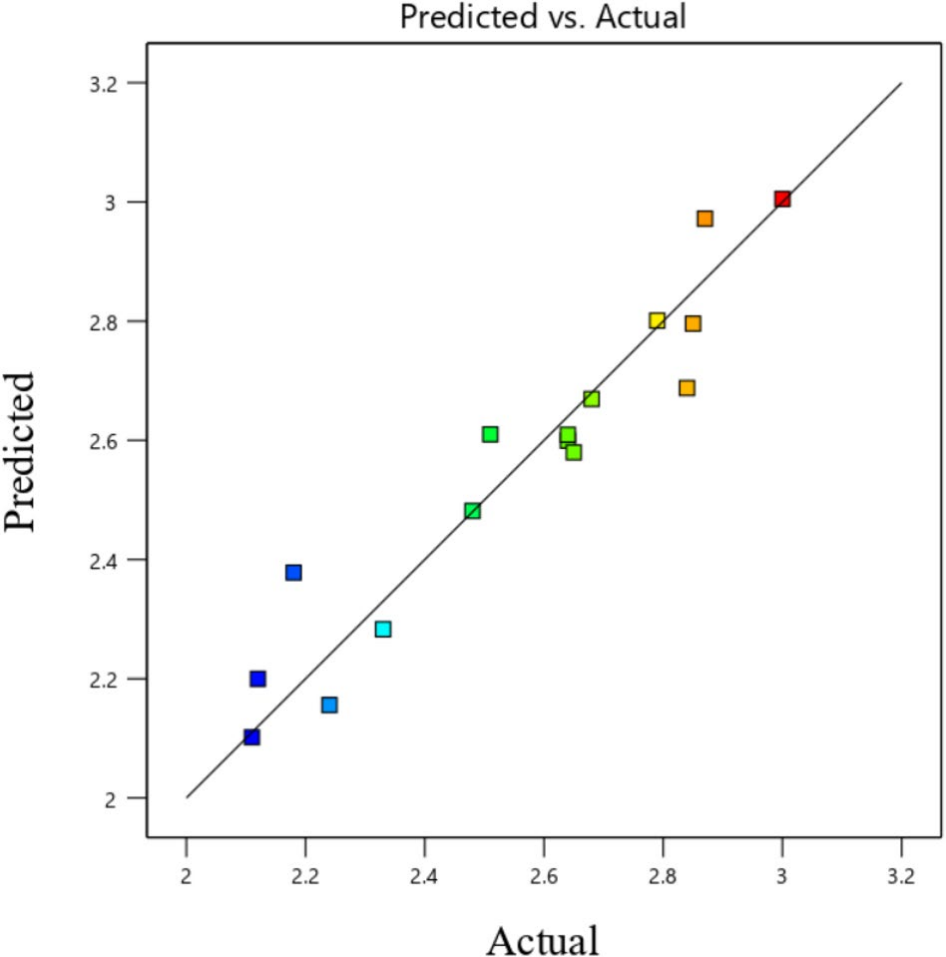
Anticipated versus real of the average Nusselt number.

ANOVA analysis of variance is used to confirm and evaluate this relationship and analyze its performance, and its results are shown in Table 3. If analysis of variance is used, it is possible to understand which factor had the greatest impact. In this table, the coefficient of determination is used to compare RSM with real data. This coefficient is a reliable indicator. This study's value reaches 0.9122, which can justify the use of RSM and confirm its agreement with the real results. Figure 26 shows the actual and predicted values for the Nu_avg_ relative to the hypothetical line. According to the obtained results, the RSM correlation estimate shows a good fit compared to the real data according to the existing complexities and the number of influencing factors. In Table 4, the optimal mode for the average Nusselt is determined by obtaining and validating the correlation so that the value of each parameter can be calculated at the optimal point. By putting these numbers in the obtained equation introduced earlier, the Nu_avg_ can be calculated, and the best optimal point is obtained.

**Table 3:** The variance analysis (ANOVA).

Source	Sum of squares	df	Mean square	F-value	*p *value	
Model	1.14	5	0.2272	20.79	< 0.0001	Significant
A-Ra	0.5523	1	0.5523	50.55	< 0.0001	
B-Ha	0.3441	1	0.3441	31.49	0.0002	
C-ε	0.1015	1	0.1015	9.29	0.0123	
D-Rd	0.0295	1	0.0295	2.70	0.1312	
E-α	0.1083	1	0.1083	9.91	0.0104	
R^2^ = 0.9122

**Table 4:** Result of the optimization strategy by RSM.

Parameter	Optimum value
Ra	1214.46
Ha	2.86
ε	0.63
Rd	0.24
α	59.35

## Conclusion

Simulation of natural convection in a corrugated trapezoidal chamber with an internal hot cavity under factors affecting the flow and heat transfer such as radiation (Rd), Rayleigh number (Ra), magnetic field angle (α), Hartmann number (Ha) and porosity (ℇ) was analyzed. Three different values were checked for each factor, considering other parameters in their average values. In this way, the effect of changing each parameter is shown more accurately and favorably. After the results obtained from the simulations and their validation, by designing the Taguchi test along with the RSM method, the optimal points for each parameter and Nuavg were determined. Also, a comprehensive relationship for calculating the Nusselt average is introduced, which provides fast and accurate results. Some of the results obtained from this research are as follows:

- It was proven that lower porosity causes higher convective heat transfer, confirmed by examining the local Nusselt diagram, so the highest Nusselt value is obtained at ℇ = 0.63.
- Convective heat transfer rate increases with the Ra number due to buoyancy force, so the Ra number and natural convection have a direct relationship, and the highest value of the Nusselt number is obtained at Ra = 1214.46.
- By applying a magnetic field, by increasing different values of Ha number, the resulting Lorentz force increases, which reduces the movement of fluid masses. Therefore, fluid flow and heat transfer inside the chamber are more effective without a magnetic field, so the maximum Nusselt number is obtained in a weak field, Ha = 2.86.
- Different amounts of radiation do not significantly affect the isotherms and flow performance inside the chamber, so the effect of this parameter can be ignored, but at Rd = 0, there is a noticeable increase in the local Nusselt number.
- An increase in the value of the angle of the magnetic field causes an increase in Nu numbers, which generally increases the natural convection so that the Nusselt number obtained at α = 59.65 is higher than other angles.
- Using the Taguchi and RSM methods, an effective relationship was presented to calculate the Nusselt average based on the determining parameters to reach an optimal point. The optimal values obtained for each of the parameters are Ra = 1214.46, Ha = 2.86, ℇ = 0.63, Rd = 0.24, and α = 59.35, and based on this, the final average Nusselt value of 3.07 was calculated.

The main objective of this study is to optimize the trapezoidal cavity with a wavy wall to maximize thermal performance and reach the optimal point for effective physical parameters. Due to the wide applications of this topic, a chosen geometry similar to the operation of a solar collector was considered here, where the fluid inside is heated by absorbing sunlight through the upper corrugated wall. Natural convection was analyzed according to the factors affecting it. Therefore, the obtained results can be used in future designs of solar collectors and engineering analyses.

More emphasis should be placed on the direction that this study will take in the future. Consequently, a deeper and more thorough understanding of the design as an input factor is possible thanks to the topological variations of enclosures with similar magnetic field, porous media, and nanoparticle parameters.

## Data Availability

The datasets used and analyzed during the current study available from the corresponding author on reasonable request.

## List of symbols

ℇ: Porosity
Rd: Radiation parameter
Ra: Rayleigh number
Ha: Hartmann number
Nu: Nusselt number
T: Temperature (k)
N: Number of waves
Cp: Specific heat capacity
g: Gravitational acceleration (m s^−^^2^)
B: Strength of magnetic field (N A^−^^1^ m^−^^2^)
x, y: Cartesian coordinates (m)
X, Y: Cartesian coordinates (dimensionless)
u,v: Components of velocities (m s^−^^1^)
U, V: Components of velocity (dimensionless)

## Greek symbols

α: Magnetic field angle (R)
β: Thermal expansion coefficient (K^−^^1^)
σ: Electrical conductivity (Ω^−^^1^ m^−^^1^)
θ: Dimensionless temperature
μ: Dynamic viscosity (kg m^−^^1^ s^−^^1^)
ρ: Density (kg m^−^^3^)
ϕ: Nanoparticles volume fraction
ψ: Dimensionless stream function
k: Thermal conductivity (W m^−^^1^ K^−^^1^

## Subscripts

nf: Nanofluid
hnf: Hybrid nanofluid
Nu_loc_: Local Nusselt number
Nu_ave_: Average Nusselt number
